# Propranolol attenuates the malignant biological behaviors of renal cancer cells partly through modulation of the IL-6/PI3K/AKT signaling axis

**DOI:** 10.3389/fphar.2026.1884603

**Published:** 2026-09-11

**Authors:** Dongming Lu, Xueping Xie, Yanmei Liu, Tianyong Zhang, Haiming Fan, Shangfan Liao, Yongyang Wu

**Affiliations:** Department of Urology, Sanming First Hospital Affiliated to Fujian Medical University, Sanming, Fujian, China

**Keywords:** drug repurposing, IL-6, PI3K/AKT, propranolol, renal cell carcinoma, β-blocker

## Abstract

**Objective:**

To investigate the effects of propranolol on the malignant biological behaviours of renal cancer cells and explore the potential involvement of the IL-6/PI3K/AKT signalling axis.

**Methods:**

786-O and Caki-2 ccRCC cells were treated with gradient propranolol, followed by CCK-8, wound-healing, Transwell and flow cytometry assays to test cell proliferation, migration, invasion and apoptosis. Western blot was applied to measure proteins associated with apoptosis, EMT, IL-6 and PI3K/AKT pathway. A xenograft nude mouse model was constructed to verify propranolol’s anti-tumor activity *in vivo*. RNA-seq combined with GO/KEGG enrichment was used to screen underlying signaling pathways. IL-6 overexpression rescue assays were performed to validate IL-6’s functional role in propranolol-mediated phenotypes.

**Results:**

Propranolol significantly inhibited the proliferation, migration and invasion of 786-O and Caki-2 cells and induced apoptosis in a concentration-dependent manner. Propranolol upregulated Bax, cleaved-Caspase-3, cleaved-Caspase-9, E-cadherin and TIMP2, while downregulating Bcl-2, N-cadherin, Vimentin and MMP9, suggesting enhanced apoptosis and suppression of EMT- and invasion-related phenotypes. *In vivo*, propranolol delayed xenograft tumour growth and reduced final tumour volume and weight. RNA-seq identified 984 differentially expressed genes, including 464 upregulated and 520 downregulated genes. Functional enrichment analysis showed that these genes were mainly associated with extracellular matrix remodelling, cytokine activity, hypoxia response, lipid metabolism and tumour-related pathways, including the PI3K/AKT signalling pathway. Western blotting confirmed that propranolol decreased the p-PI3K/PI3K and p-AKT/AKT ratios. In addition, propranolol reduced IL-6 protein expression in renal cancer cells, whereas IL-6 overexpression partially restored propranolol-suppressed cell proliferation, migration and invasion, and partially reversed propranolol-induced changes in apoptosis-related proteins, EMT-related proteins and PI3K/AKT pathway activation.

**Conclusion:**

Propranolol suppresses the malignant biological behaviours of renal cancer cells and inhibits renal tumour growth *in vivo*. These effects may be mediated, at least in part, through downregulation of IL-6 and suppression of PI3K/AKT signalling pathway activation.

## 1 Introduction

Renal cell carcinoma (RCC) is one of the most common malignancies of the urinary system. Clear cell renal cell carcinoma (ccRCC) is the predominant histological subtype, accounting for approximately 75%–80% of all RCC cases. Standard first-line regimens include anti-angiogenic targeted drugs and immune checkpoint inhibitors, yet many patients develop secondary resistance within months of treatment. Despite recent advances in surgery, molecular targeted therapy and immunotherapy, patients with advanced or metastatic RCC continue to face a high risk of recurrence and metastasis, therapeutic resistance and poor long-term outcomes. In addition, expensive novel agents carry substantial toxic side effects that limit long-term clinical application. Therefore, further elucidation of the molecular mechanisms underlying RCC progression and the exploration of novel therapeutic strategies with translational potential remain of considerable importance.

In recent years, increasing evidence has indicated that stress-related neuroendocrine signalling plays an important regulatory role in tumour initiation and progression. Under conditions of chronic stress, sustained catecholamine release can activate β-adrenergic receptors (β-ARs), thereby contributing to the regulation of multiple malignant biological behaviours, including tumour cell proliferation, resistance to apoptosis, angiogenesis, inflammatory responses, invasion and metastasis (Fjæstad et al., 2022; O'Logbon et al., 2025). Propranolol is a classical non-selective β-adrenergic receptor blocker that has been widely used in cardiovascular diseases and has a well-established safety profile. In recent years, based on the concept of drug repurposing, the potential value of propranolol in cancer therapy has attracted increasing attention (Albiñana et al., 2020).

Previous studies have suggested that propranolol may exert antitumour activity in clear cell renal cell carcinoma (Albiñana et al., 2020; Albiñana et al., 2022). Albiñana et al. reported that propranolol induced apoptosis in renal cancer cells by modulating HIF-2α and its downstream targets, including VEGF, CAIX and AQP-1 (Albiñana et al., 2020). Another study showed that propranolol reduced intracellular reactive oxygen species (ROS) levels and inhibited NF-κB/p65-related inflammatory signalling, thereby delaying tumour growth (Albiñana et al., 2022). These findings suggest that propranolol may regulate renal cancer progression through multiple targets and signalling pathways. However, its precise mechanism of action in RCC remains to be further elucidated.

The PI3K/AKT signalling pathway is one of the key signalling axes closely associated with tumour cell proliferation, survival, migration, invasion and therapeutic resistance in RCC, particularly in ccRCC (Sato et al., 2013; Zhang et al., 2024). In addition, studies in other tumour types have suggested a potential association between propranolol and the regulation of PI3K/AKT signalling (Hu et al., 2021). However, direct experimental evidence showing whether propranolol suppresses the malignant biological behaviours of renal cancer cells through modulation of the PI3K/AKT signalling pathway remains limited.

Interleukin-6 (IL-6) has been implicated in cancer cell proliferation, survival, migration, invasion and therapeutic resistance. Previous studies have suggested that β2-adrenergic receptor blockade can attenuate oxidative stress and inflammatory responses in ccRCC models, whereas IL-6-related signalling can activate downstream pathways such as PI3K/AKT and promote ccRCC progression (Albiñana et al., 2022; Lu et al., 2024). Therefore, IL-6 may represent a potential downstream mediator connecting propranolol treatment with PI3K/AKT pathway regulation in renal cancer.

Based on these findings, this study aimed to systematically evaluate the effects of propranolol on renal cancer cell proliferation, migration, invasion, apoptosis and tumour-forming ability *in vivo*. By integrating RNA sequencing, functional enrichment analysis, Western blot validation and IL-6 overexpression rescue experiments, we further explored the potential involvement of the IL-6/PI3K/AKT signalling axis in the antitumour effects of propranolol in renal cancer. This study may provide experimental evidence and theoretical support for the repurposing of propranolol as a potential therapeutic strategy for RCC.

## 2 Materials and methods

### 2.1 Cell lines and culture

Human renal cell carcinoma cell lines 786-O and Caki-2 were obtained from the Cell Bank of the Chinese Academy of Sciences (Shanghai, China). Cells were cultured in high-glucose Dulbecco’s modified Eagle’s medium (DMEM; Thermo Fisher Scientific, Waltham, MA, USA) supplemented with 10% fetal bovine serum (FBS; Thermo Fisher Scientific, Waltham, MA, USA) and 1% penicillin/streptomycin solution (Gibco, Grand Island, NY, USA). Cells were maintained in a humidified incubator at 37 °C with 5% CO_2_. The culture medium was replaced every 48 h, and cells in the logarithmic growth phase were used for subsequent experiments.

### 2.2 Reagents and antibodies

Propranolol hydrochloride was purchased from MedChemExpress (MCE, USA). A stock solution was prepared in dimethyl sulfoxide (DMSO; Sangon Biotech, Shanghai, China) and diluted with complete culture medium to the required final concentrations. The final concentration of DMSO was kept consistent across all treatment groups.

The Cell Counting Kit-8 (CCK-8) assay kit was purchased from Dojindo Molecular Technologies (Kumamoto, Japan). TRIzol reagent was purchased from Invitrogen (Carlsbad, CA, USA). RIPA lysis buffer and the BCA protein assay kit were purchased from Thermo Fisher Scientific (Waltham, MA, USA). Enhanced chemiluminescence (ECL) reagent, electrophoresis buffer and transfer buffer were purchased from Bio-Rad (Hercules, CA, USA). The Annexin V-FITC/PI apoptosis detection kit was purchased from Beyotime Biotechnology (Shanghai, China). Matrigel matrix and Transwell chambers were purchased from Corning (Corning, NY, USA).

The primary antibodies used for Western blotting included antibodies against IL-6,Bcl-2, Bax, cleaved-Caspase-3, cleaved-Caspase-9, E-cadherin, N-cadherin, Vimentin, TIMP2, MMP9 and GAPDH. Antibodies used for detection of the PI3K/AKT signalling pathway included PI3K, p-PI3K, AKT and p-AKT. HRP-conjugated secondary antibodies were also used. The primary antibodies used for Western blotting were purchased from Abcam (Cambridge, UK) or Proteintech (Wuhan, China), as indicated below.

### 2.3 Cell treatment

786-O and Caki-2 cells were treated with propranolol at final concentrations of 0, 10, 20 and 50 μM. The control group was treated with an equal volume of vehicle. According to the specific experimental design, cells were subsequently collected or analysed for proliferation, migration, invasion, apoptosis and related protein expression after propranolol treatment.

### 2.4 IL-6 overexpression and rescue experiments

To investigate whether IL-6 is involved in the biological effects of propranolol on renal cancer cells, IL-6 overexpression rescue experiments were performed. 786-O and Caki-2 cells were transfected with an IL-6 overexpression plasmid or the corresponding empty vector using Lipofectamine 3000 transfection reagent according to the manufacturer’s instructions. After transfection for 24–48 h, IL-6 overexpression efficiency was verified by Western blot analysis. For rescue experiments, cells were divided into four groups: control, propranolol treatment, IL-6 overexpression, and propranolol + IL-6 overexpression. After IL-6 overexpression was established, cells in the propranolol and propranolol + IL-6 overexpression groups were treated with 20 μM propranolol. This intermediate concentration was selected to produce a measurable inhibitory effect while avoiding excessive suppression of cell viability and other malignant phenotypes, thereby allowing sufficient experimental scope to evaluate the potential rescuing effect of IL-6 overexpression. Cells were then collected for subsequent CCK-8, wound-healing, Transwell invasion and Western blot assays.

### 2.5 Cell viability assay

Cell viability was assessed using the Cell Counting Kit-8 (CCK-8) assay. 786-O and Caki-2 cells were seeded into 96-well plates at a density of 2 × 10^3^ cells per well in 100 μL of complete medium, with three replicate wells set up for each group. After cell attachment, the cells were treated with different concentrations of propranolol. At 0, 24, 48 and 72 h after treatment, 10 μL of CCK-8 reagent was added to each well, followed by incubation at 37 °C for 4 h. The absorbance at 450 nm was then measured using a microplate reader (Varioskan ALF, Thermo Fisher Scientific, USA). Cell viability was expressed as the optical density value.

### 2.6 Apoptosis assay

Cell apoptosis was detected using Annexin V-FITC/PI double staining followed by flow cytometry. 786-O and Caki-2 cells were treated with propranolol at concentrations of 0, 10, 20 and 50 μM for 24 h. Cells were then digested with EDTA-free trypsin, collected by centrifugation, washed twice with pre-cooled PBS and resuspended in binding buffer. Annexin V-FITC and PI were added according to the manufacturer’s instructions, and the cells were incubated at room temperature in the dark for 15 min. Apoptosis was then analysed using a flow cytometer (BD Fortessa X20, BD Biosciences, USA). The sum of early and late apoptotic cells was defined as the total apoptosis rate.

### 2.7 Transwell invasion assay

Cell invasion was assessed using Matrigel-precoated Transwell chambers with 8.0-μm pore membranes. Matrigel was diluted with serum-free medium at a ratio of 1:8 and added to the upper chambers. After serum starvation, cells were harvested and resuspended in serum-free medium at a density of 5 × 10^5^ cells/mL. A total of 200 μL of the cell suspension was added to the upper chamber, while 500 μL of complete medium was added to the lower chamber. After incubation for 48 h, non-invading cells remaining on the upper surface of the membrane were gently removed. Cells that had invaded through the membrane to the lower surface were fixed, stained with crystal violet, photographed under a microscope and counted.

### 2.8 Wound-healing assay

Cell migration was evaluated using a wound-healing assay. Cells were seeded into 6-well plates and cultured until they reached near confluence. A straight scratch was then made in the centre of the cell monolayer using a 200-μL pipette tip, followed by gentle washing with PBS to remove detached cells. Images were captured under a microscope at 0 h. The cells were then cultured in serum-free medium for 24 h, and images of the same fields were captured again. Cell migration ability was assessed by calculating the wound closure rate.

### 2.9 Western blot analysis

Following drug intervention, total cellular protein was extracted using RIPA lysis buffer supplemented with protease inhibitors. Cell lysates were subjected to low-temperature centrifugation, and supernatants were collected for protein quantification using a BCA protein assay kit. Equal protein aliquots (40 μg per lane) were separated via SDS-PAGE and electrotransferred onto PVDF membranes.

Subsequently, the membranes were blocked with 5% non-fat milk at room temperature for 2 h and incubated overnight at 4 °C with primary antibodies. The primary antibodies used for Western blotting were purchased from Abcam (Cambridge, UK) or Proteintech (Wuhan, China). Antibodies with catalogue numbers beginning with “ab” were purchased from Abcam, whereas the IL-6 antibody was purchased from Proteintech. The detailed antibody information is listed as follows: IL-6 (21865-1-AP, 1:2000), Bcl-2 (ab182858, 1:2000), Bax (ab32503, 1:1000), cleaved Caspase-3 (ab32042, 1:1000), cleaved Caspase-9 (ab214430, 1:1000), E-cadherin (ab40772, 1:1000), N-cadherin (ab76011, 1:1000), Vimentin (ab92547, 1:1000), TIMP2 (ab180630, 1:1000), MMP9 (ab76003, 1:1000), and GAPDH (ab181602, 1:1000). After thorough washing with TBST, the blots were incubated with HRP-conjugated secondary antibodies at room temperature for 1 h. Protein bands were visualized using enhanced chemiluminescence (ECL) reagent and captured with a Tanon 5200 Multi chemiluminescence imaging system. GAPDH was used as an internal loading control. Band intensities were quantified by ImageJ software and normalized to GAPDH. All experiments were performed in biological triplicate. To evaluate the activation of the PI3K/AKT signaling pathway, the expression levels of total and phosphorylated PI3K and AKT were detected by Western blot. The primary antibodies included p-PI3K (ab278545, 1:1000), PI3K (ab302958, 1:1000), p-AKT (ab81283, 1:1000), AKT (ab8805, 1:1000), and β-actin (ab8227, 1:1000), with β-actin serving as the loading control. The activation level of the PI3K/AKT pathway was calculated as the relative ratios of p-PI3K/PI3K and p-AKT/AKT. For IL-6 rescue experiments, Western blot was performed to detect the expression of IL-6, apoptosis-related proteins, EMT and metastasis-associated proteins, as well as PI3K/AKT signaling pathway proteins in four experimental groups: control group, propranolol single treatment group, IL-6 overexpression group, and propranolol combined with IL-6 overexpression group. The phosphorylation levels of PI3K and AKT were quantified via the aforementioned ratio calculation method to assess pathway activation.

### 2.10 Animal experiments

Specific pathogen-free (SPF) male BALB/c nude mice (n = 6/group), aged 4–6 weeks and weighing 20–25 g, were purchased from Jiangsu Jicui Yaokang Biotechnology Co., Ltd. The mice were housed under SPF conditions at a temperature of 22 °C ± 1 °C and a relative humidity of 65%–70%, with a 12-h light/dark cycle and free access to food and water.

After 7 days of acclimatisation, 786-O cells were harvested and prepared as a single-cell suspension. Each mouse was subcutaneously inoculated with 1 × 10^7^ cells in 200 μL into the left dorsal flank. When the tumour volume reached approximately 50–100 mm^3^, the mice were randomly divided into a control group and a propranolol-treated group, with six mice in each group. Propranolol was administered by intraperitoneal injection at a dose of 10 mg/kg/day for 15 consecutive days, starting on day 7 after cell inoculation when tumours had formed.

During the experiment, tumour length (a) and width (b) were measured every 3 days, and tumour volume was calculated using the formula: V = 0.5 × a × b^2^. Tumour growth curves were then plotted. After 15 days of treatment and observation, the mice were euthanised, and the tumour tissues were excised, photographed and weighed. A portion of the tumour tissues was fixed, embedded and sectioned for immunohistochemical detection of Ki-67 expression. Another portion of each tumour tissue was immediately snap-frozen in liquid nitrogen and stored at −80 °C until total RNA extraction and qPCR analysis.

All animal experiments were approved by the Animal Ethics Committee of Sanming First Hospital, with the approval number **(C2025) 207**.

### 2.11 Quantitative real-time PCR analysis of xenograft tumour tissues

Quantitative real-time PCR (qRT-PCR) was performed to detect the relative mRNA expression levels of AKT, PI3K, IL-6, E-cadherin and Bax in xenograft tumour tissues. A total of 12 xenograft tumour samples were included, with six samples from the control group and six samples from the propranolol-treated group. Total RNA was extracted using the RNeasy Mini Kit (QIAGEN, Cat. No. 74104) according to the manufacturer’s instructions. RNA concentration and quality were assessed using a One Drop spectrophotometer. cDNA was synthesized using a First Strand cDNA Synthesis Kit (Beyotime, Cat. No. D7190L). qRT-PCR was performed using 2×RealStar Fast SYBR qPCR Mix (Kangrun Chengye Biotechnology, Cat. No. A301-10) on a real-time fluorescence quantitative PCR system (BIOER, China). Each 20 μL reaction mixture contained 10 μL of 2×RealStar Fast SYBR qPCR Mix, 1 μL of forward primer, 1 μL of reverse primer, 1 μL of cDNA template and nuclease-free water to a final volume of 20 μL. The PCR cycling conditions were as follows: initial denaturation at 95 °C for 5 min, followed by 40 cycles of denaturation at 95 °C for 15 s and annealing/extension at 60 °C for 15 s. GAPDH was used as the internal reference gene, and relative mRNA expression levels were calculated using the 2^-ΔΔCt^ method. Primer sequences are listed in Supplementary Table S1.

### 2.12 RNA sequencing and bioinformatics analysis

Control and propranolol-treated 786-O cells were subjected to RNA-seq analysis, with three biological replicates included in each group. The samples were designated as NC-1, NC-2, NC-3 and Treat-1, Treat-2, Treat-3, respectively. Total RNA was extracted using TRIzol reagent, and RNA concentration, purity and integrity were assessed. After quality control, qualified RNA samples were used for library construction, library purification, library quality assessment and library quantification, followed by paired-end sequencing on an Illumina sequencing platform.

Raw sequencing data were converted into FASTQ files after base calling using Bcl2fastq. Sequencing quality was assessed using FastQC, and raw reads were filtered using Fastp to remove adaptor sequences, low-quality bases and reads with insufficient length, thereby generating clean reads. After filtering, the Q30 values of all samples remained at a high level, indicating good sequencing quality.

Clean reads were subsequently aligned to the human reference genome hg38/GRCh38 using HISAT2 v2.2.1. The overall mapping rate of clean reads was approximately 98% across all samples, with a unique mapping rate of approximately 95%. Gene expression levels were quantified using HTSeq and expressed as fragments per kilobase of transcript per million mapped reads (FPKM).

Differential expression analysis was performed using DESeq2. The Benjamini–Hochberg false discovery rate (FDR) method was applied for multiple testing correction to calculate adjusted P values. Differentially expressed genes were identified using the criteria of |log_2_FC| > 1.2 and adjusted P < 0.05. Gene Ontology (GO) functional enrichment analysis and Kyoto Encyclopedia of Genes and Genomes (KEGG) pathway enrichment analysis were subsequently performed for the differentially expressed genes.

### 2.13 Statistical analysis

All *in vitro* assays were repeated in at least three independent biological replicates, with six nude mice assigned to each *in vivo* group; all data are expressed as mean ± SD, and statistical analyses were performed using GraphPad Prism 9.0. Normality was assessed using the Shapiro–Wilk test before parametric analyses where applicable. Considering the limited sample size of some *in vitro* assays, the statistical results were interpreted cautiously. Comparisons between two groups were performed using an unpaired two-tailed Student’s t-test. Comparisons among multiple groups were performed using one-way ANOVA followed by Tukey’s *post hoc* test. Cell viability data involving different drug concentrations and time points were analysed using two-way ANOVA, and xenograft tumour growth curves were compared using two-way repeated-measures ANOVA. Western blot band intensities were quantified after normalization to internal reference proteins, while IHC results were assessed by positive staining proportion or mean optical density. All statistical tests were two-tailed, and P < 0.05 was regarded as statistically significant.

## 3 Results

### 3.1 Propranolol inhibits proliferation, migration and invasion and induces apoptosis in renal cancer cells

**FIGURE 1:**
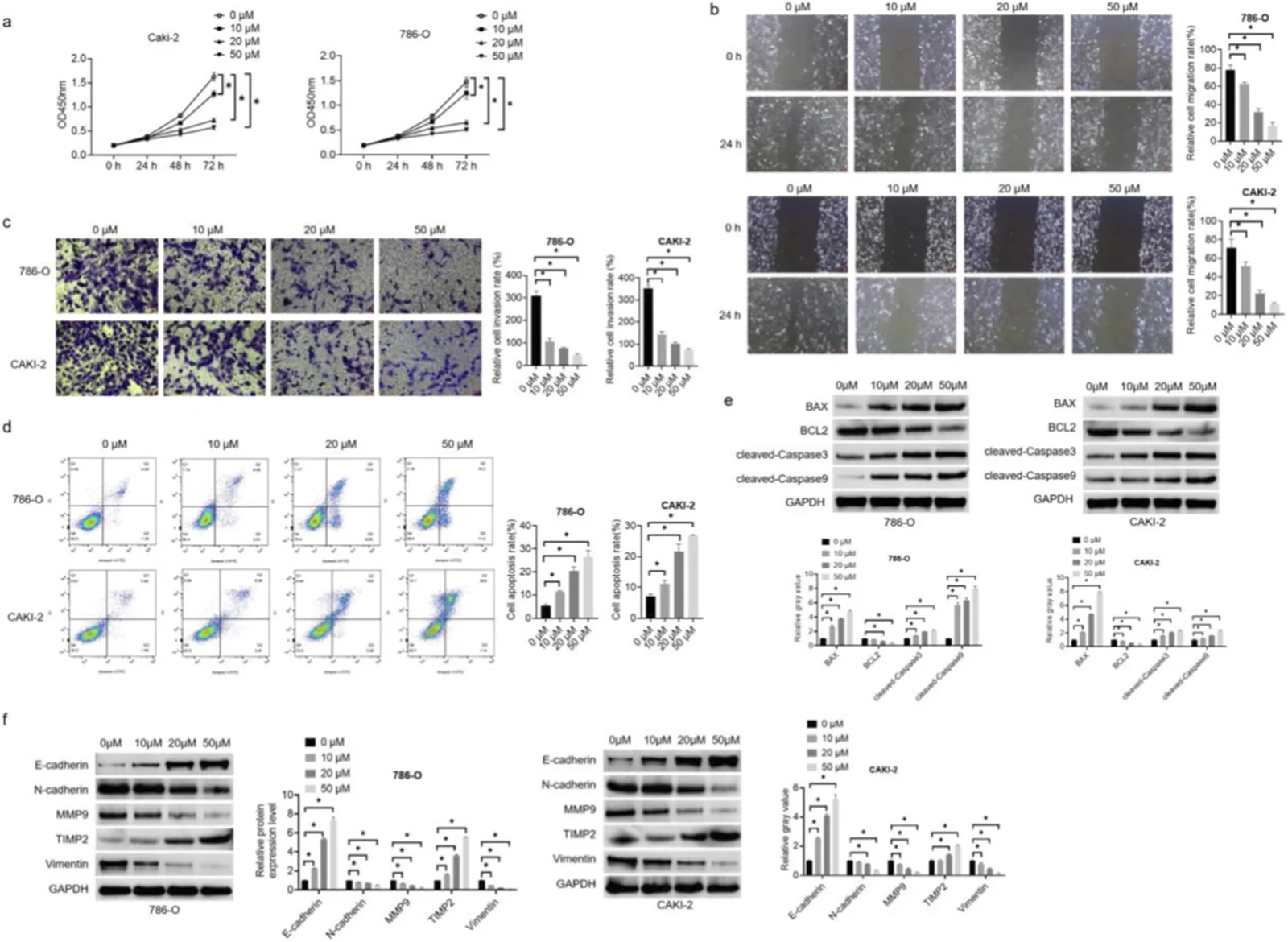
Propranolol suppresses malignant phenotypes and regulates apoptosis- and metastasis-related protein expression in renal cell carcinoma cells. **(a)** Cell viability of 786-O and Caki-2 cells treated with propranolol at 0, 10, 20, and 50 μM was measured by CCK-8 assay at 0, 24, 48, and 72 h. **(b)** Representative wound-healing images of 786-O and Caki-2 cells treated with 0, 10, 20, and 50 μM propranolol. Images were captured at 0 and 24 h after scratching. **(c)** Transwell invasion assay of 786-O and Caki-2 cells treated with 0, 10, 20, and 50 μM propranolol. Invaded cells were fixed, stained with crystal violet, and imaged after 48 h of incubation. **(d)** Flow cytometric analysis of apoptosis in 786-O and Caki-2 cells treated with 0, 10, 20, and 50 μM propranolol for 24 h using Annexin V-FITC/PI double staining. **(e)** Western blot analysis of apoptosis-related proteins, including BAX, BCL-2, cleaved-Caspase-3, and cleaved-Caspase-9, in 786-O and Caki-2 cells treated with 0, 10, 20, and 50 μM propranolol. GAPDH was used as the loading control. **(f)** Western blot analysis of EMT- and invasion/metastasis-related proteins, including E-cadherin, N-cadherin, Vimentin, MMP9, and TIMP2, in 786-O and Caki-2 cells treated with 0, 10, 20, and 50 μM propranolol. GAPDH was used as the loading control. All data are presented as the mean ± SD from three independent experiments. Statistical significance was determined by one-way or two-way ANOVA followed by Tukey’s multiple-comparisons test. Data are shown as mean ± SD, n = 3 independent biological replicates. *P < 0.05, **P < 0.01.

To evaluate the effects of propranolol on the malignant biological behaviour of renal cancer cells, *in vitro* functional assays were performed using two renal cancer cell lines, 786-O and Caki-2. Cell proliferation was first assessed using the CCK-8 assay. Propranolol treatment significantly reduced the proliferative activity of both 786-O and Caki-2 cells, with a progressively stronger inhibitory effect observed as the drug concentration increased, indicating that propranolol suppresses renal cancer cell proliferation in a dose-dependent manner (Figure 1a).

Cell migration was further evaluated using a wound-healing assay. Compared with the control group, propranolol treatment markedly reduced wound closure in both 786-O and Caki-2 cells, and this inhibitory effect became more pronounced with increasing drug concentrations. These results indicate that propranolol effectively suppresses the migratory capacity of renal cancer cells (Figure 1b).

Cell invasion was subsequently assessed using Transwell assays. Propranolol treatment significantly reduced the invasive capacity of both 786-O and Caki-2 cells in a dose-dependent manner, as reflected by a progressive decrease in the number of cells invading through the membrane with increasing propranolol concentrations. These findings indicate that propranolol suppresses the invasive phenotype of renal cancer cells (Figure 1c).

To further determine the effect of propranolol on apoptosis, Annexin V-FITC/PI double-staining flow cytometry was performed. Propranolol treatment significantly induced apoptosis in both 786-O and Caki-2 cells, with early apoptosis, late apoptosis and total apoptosis rates increasing progressively as the drug concentration increased, suggesting a dose-dependent pro-apoptotic effect (Figure 1d).

Western blot analysis was then performed to examine changes in apoptosis-related proteins. Propranolol treatment upregulated the expression of the pro-apoptotic proteins Bax, cleaved-Caspase-3 and cleaved-Caspase-9, while downregulating the anti-apoptotic protein Bcl-2, with these changes showing a dose-dependent pattern (Figure 1e). Collectively, these results indicate that propranolol promotes apoptosis in renal cancer cells.

Western blot analysis was performed to evaluate the effects of propranolol on the expression of EMT- and invasion/metastasis-related proteins. With increasing concentrations of propranolol, the expression of the epithelial marker E-cadherin and the matrix metalloproteinase inhibitor TIMP2 was progressively upregulated in both 786-O and Caki-2 cells, whereas the expression of the mesenchymal markers N-cadherin and Vimentin, as well as the invasion-related protein MMP9, was gradually downregulated in a dose-dependent manner (Figure 1f). These findings suggest that propranolol suppresses EMT progression and invasion/metastasis-related phenotypes in renal cancer cells.

### 3.2 Propranolol suppresses renal tumour growth in a nude mouse xenograft model

**FIGURE 2:**
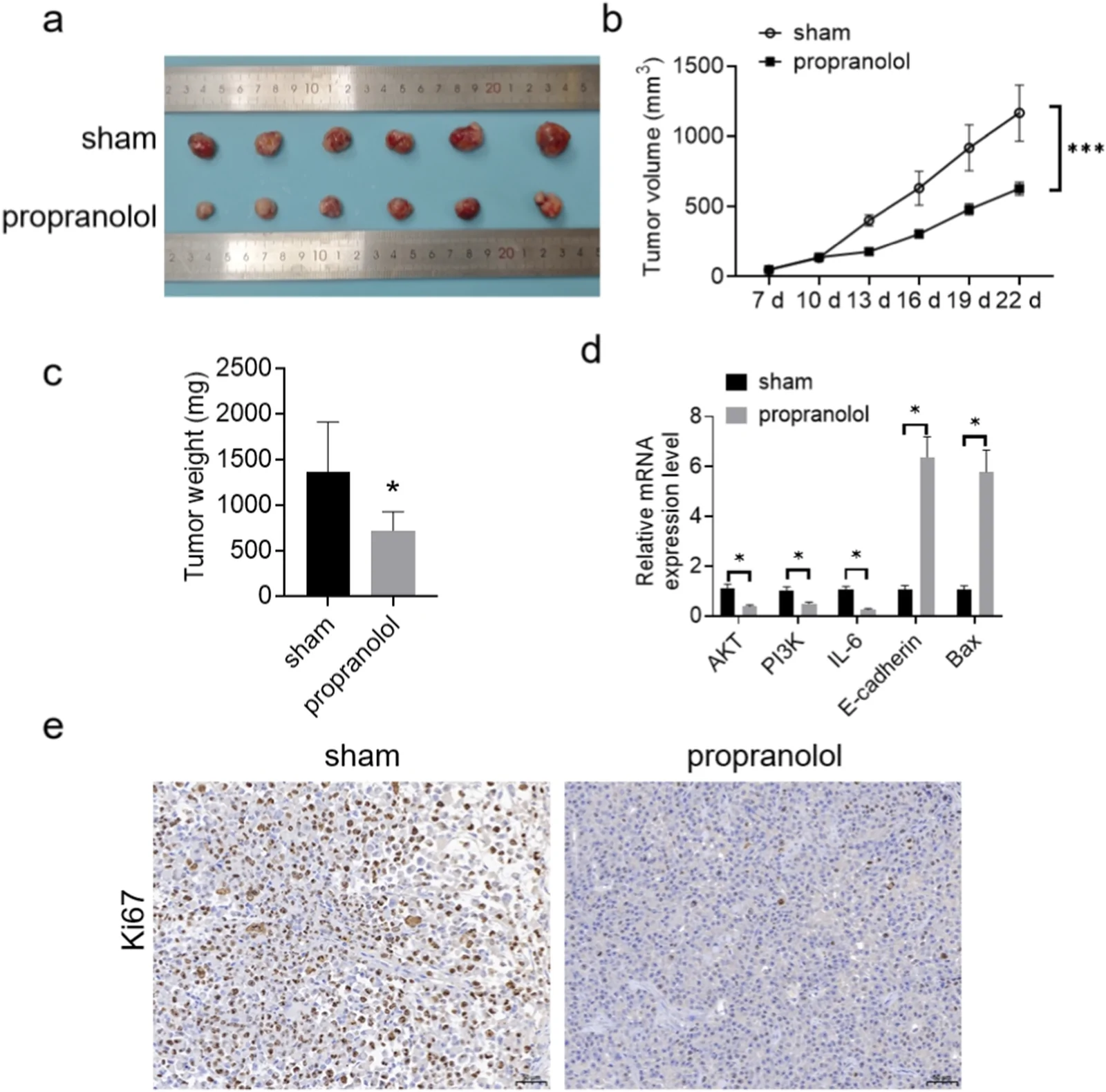
Propranolol suppresses the growth of renal cancer subcutaneous xenografts in nude mice. **(a)** Representative images of excised xenograft tumours from each group. **(b)** Growth curves of subcutaneous xenograft tumours showing changes in tumour volume over time in each group. **(c)** Quantitative analysis of tumour weight in each group. **(d)** Relative mRNA expression of AKT, PI3K, IL-6, E-cadherin and Bax in xenograft tumor tissues detected by qPCR. **(e)** Immunohistochemical detection of Ki-67 expression in xenograft tumour tissues. Data are presented as the mean ± SD. n = 6 mice per group. *P < 0.05, **P < 0.01, ***P < 0.001.

To further validate the antitumour activity of propranolol *in vivo*, a subcutaneous xenograft model was established using 786-O cells in nude mice. Compared with the control group, propranolol treatment markedly delayed xenograft tumour growth, as reflected by a significantly lower tumour growth curve in the propranolol-treated group (Figures 2a,b). At the experimental endpoint, excised tumours from the propranolol-treated mice showed a significant reduction in tumour weight compared with those from control mice (Figure 2c), indicating that propranolol effectively suppresses renal cancer xenograft growth *in vivo*. qPCR analysis of xenograft tumor tissues revealed that propranolol significantly downregulated the mRNA levels of AKT, PI3K and IL-6, while markedly upregulated E-cadherin and Bax expression (P < 0.05 *versus* the sham group (Figure 2d)). Further immunohistochemical analysis demonstrated that the expression of Ki-67, a proliferation marker, was markedly decreased in xenograft tissues from the propranolol-treated group compared with the control group (Figure 2e), suggesting that propranolol inhibits the proliferative activity of tumour cells *in vivo*. Collectively, these findings demonstrate that propranolol exerts a robust antitumour effect in a 786-O cell-derived subcutaneous xenograft model.

### 3.3 Propranolol suppresses phosphorylation-mediated activation of the PI3K/AKT signalling pathway

**FIGURE 3:**
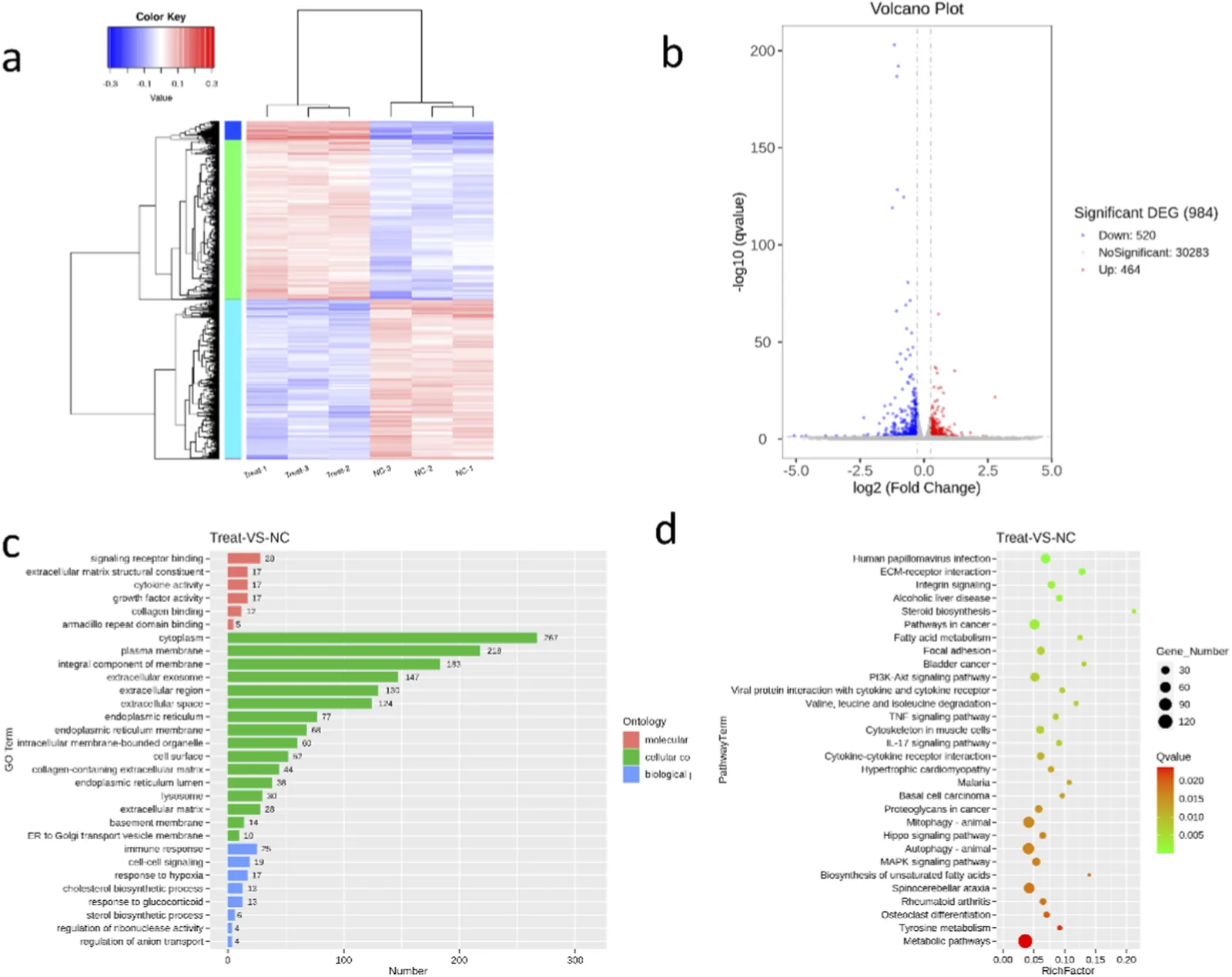
Propranolol-induced transcriptomic alterations and functional enrichment landscape in renal cancer cells. **(a)** Hierarchical clustering heatmap of differentially expressed genes between the propranolol-treated group (Treat) and the control group (NC). **(b)** Volcano plot of differentially expressed genes between the propranolol-treated group and the control group. **(c)** GO functional enrichment analysis of differentially expressed genes. **(d)** KEGG pathway enrichment analysis of differentially expressed genes.

To investigate the effect of propranolol on the gene expression profile of renal cancer cells, RNA sequencing was performed in the propranolol-treated group (Treat) and the control group (NC). Hierarchical clustering of the RNA-seq data showed clear separation between the Treat and NC samples, with good consistency among biological replicates within each group (Figure 3a), indicating that propranolol treatment induced substantial alterations in the global transcriptomic profile of renal cancer cells.

Differential expression analysis further identified 984 differentially expressed genes in the Treat group compared with the NC group, including 464 upregulated and 520 downregulated genes (Figure 3b). The volcano plot demonstrated a distinct distribution of differentially expressed genes between the two groups, suggesting that propranolol treatment induces broad transcriptomic remodelling in renal cancer cells.

GO and KEGG enrichment analyses showed that the differentially expressed genes were mainly associated with biological processes related to extracellular matrix remodelling, cytokine activity, response to hypoxia and lipid metabolism, and were significantly enriched in pathways including the PI3K-Akt signalling pathway, ECM-receptor interaction, focal adhesion, cytokine-cytokine receptor interaction and metabolic pathways (Figures 3c,d). These findings suggest that the transcriptional changes induced by propranolol are predominantly involved in tumour-related signal transduction, extracellular matrix remodelling, inflammatory cytokine networks and lipid metabolic pathways.

### 3.4 Propranolol suppresses activation of the PI3K/AKT signalling pathway in renal cancer cells

**FIGURE 4:**
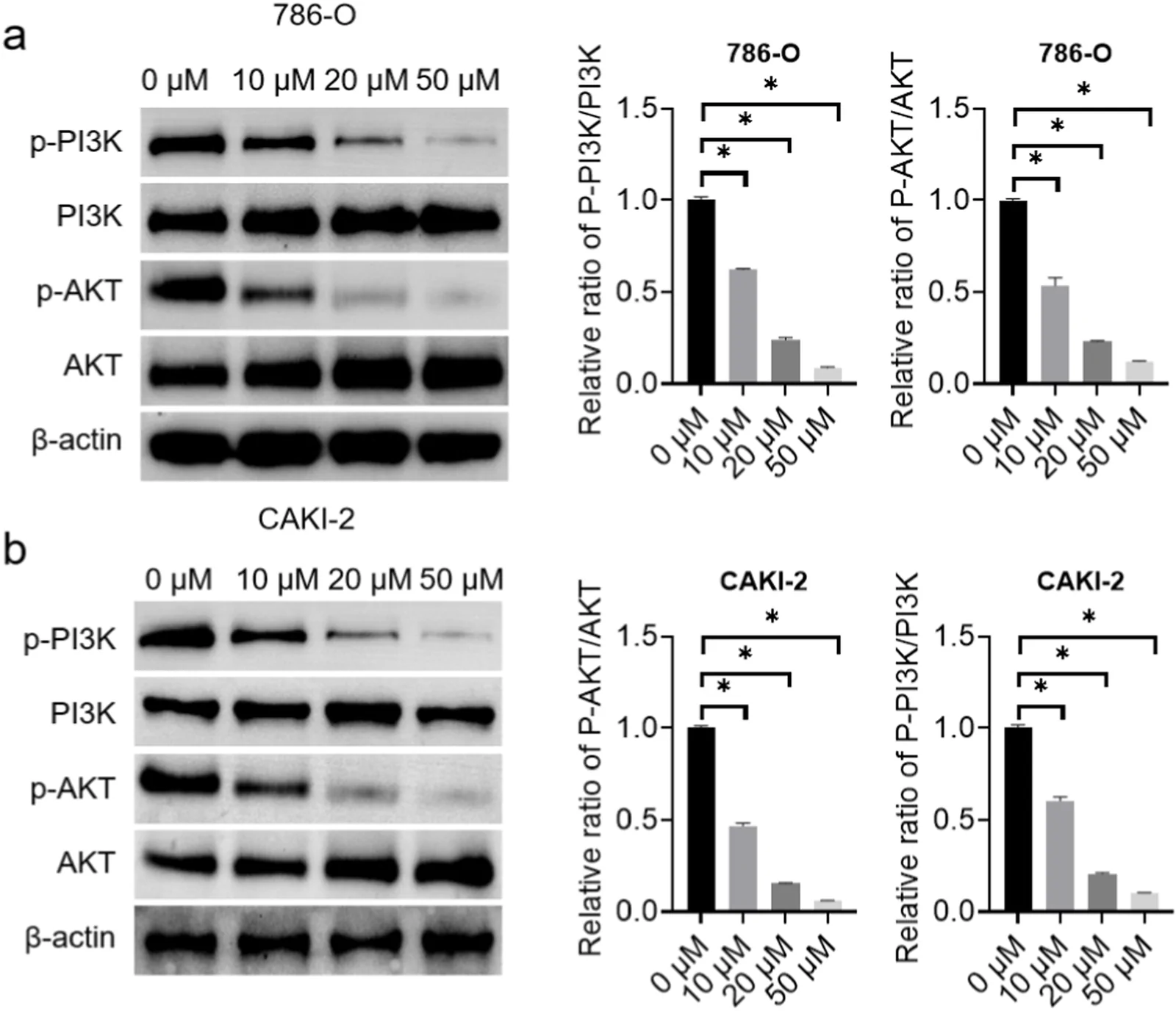
Propranolol inhibits activation of the PI3K/AKT signalling pathway in renal cancer cells. **(a)** Western blot analysis of PI3K/AKT signalling-related proteins in 786-O and Caki-2 renal cancer cells treated with different concentrations of propranolol. **(b)** Quantitative analysis of the relative p-PI3K/PI3K and p-AKT/AKT ratios. β-actin was used as the loading control. Data are shown as mean ± SD, n = 3 independent biological replicates. *P < 0.05.

Western blot analysis was further performed to assess the phosphorylation levels and total protein expression of key components of the PI3K/AKT signalling pathway (Figure 4). The results showed that the ratios of p-PI3K/PI3K and p-AKT/AKT decreased significantly with increasing concentrations of propranolol, indicating that propranolol suppresses the phosphorylation-mediated activation of PI3K and AKT. Together with the preceding functional assays and transcriptomic enrichment analyses, these findings suggest that the PI3K/AKT signalling pathway may represent one of the key molecular mechanisms underlying the antitumour activity of propranolol in renal cancer.

### 3.5 IL-6 overexpression partially restores the inhibitory effects of propranolol on renal cancer cell proliferation, migration, and invasion

**FIGURE 5:**
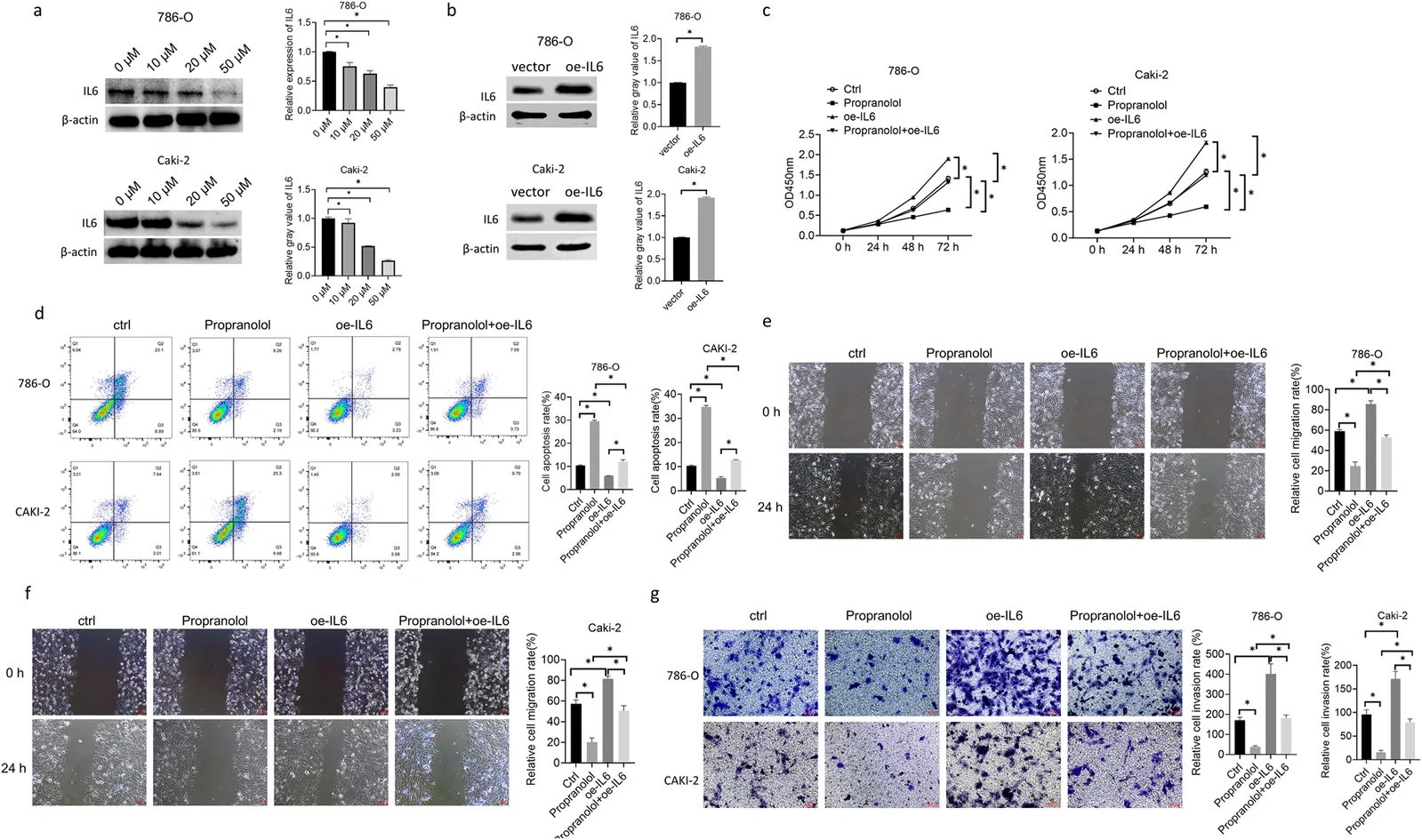
Propranolol inhibits the malignant biological behaviours of renal cancer cells through the downregulation of IL-6 expression. **(a)** Western blot analysis was performed to detect IL-6 protein expression in 786-O and Caki-2 renal cancer cells after treatment with different concentrations of propranolol. **(b)** Western blot analysis was performed to detect IL-6 protein expression in 786-O and Caki-2 cells after IL-6 overexpression. **(c)** CCK-8 assays were performed to evaluate the proliferative activity of 786-O and Caki-2 cells in the control, propranolol, IL-6 overexpression, and propranolol + IL-6 overexpression groups. **(d)** Flow cytometric analysis of apoptosis in 786-O and Caki-2 cells using Annexin V-FITC/PI double staining. **(e,f)** Wound healing assays were performed to assess the migratory ability of 786-O and Caki-2 cells in the control, propranolol, IL-6 overexpression, and propranolol + IL-6 overexpression groups. **(g)** Transwell invasion assays were performed to evaluate the invasive ability of 786-O and Caki-2 cells in the control, propranolol, IL-6 overexpression, and propranolol + IL-6 overexpression groups. Data are presented as the mean ± SD, n = 3 independent biological replicates. *P < 0.05.

To further explore the potential downstream factors involved in the antitumour effects of propranolol, we examined IL-6 protein expression in renal cancer cells after propranolol treatment. Western blot analysis showed that IL-6 protein expression gradually decreased in 786-O and Caki-2 cells as the concentration of propranolol increased (Figure 5a). To determine whether IL-6 is involved in the biological effects of propranolol, IL-6 overexpression rescue experiments were subsequently performed. Western blot analysis showed that IL-6 protein expression was markedly increased in the IL-6 overexpression group compared with the vector control group (Figure 5b).

Further functional assays showed that propranolol significantly inhibited the proliferative activity of 786-O and Caki-2 cells, whereas IL-6 overexpression enhanced cell proliferation. Compared with the propranolol treatment group, the propranolol + IL-6 overexpression group showed a partial restoration of cell proliferative activity (Figure 5c). Cell apoptosis was promoted by propranolol treatment, whereas IL-6 overexpression inhibited cell apoptosis. Compared with the propranolol treatment group, the propranolol + IL-6 overexpression group showed a partial restoration of cell apoptosis activity (Figure 5d). Wound healing assays showed that propranolol treatment markedly reduced the migratory ability of 786-O and Caki-2 cells, whereas IL-6 overexpression promoted cell migration. Compared with the propranolol treatment group, the propranolol + IL-6 overexpression group showed a partial recovery of wound closure ability (Figures 5e,f). Transwell invasion assays showed that propranolol significantly reduced the invasive ability of 786-O and Caki-2 cells, whereas IL-6 overexpression enhanced cell invasion. Compared with the propranolol group, the number of invading cells was partially restored in the propranolol + IL-6 overexpression group (Figure 5g).

### 3.6 IL-6 overexpression partially reverses propranolol-induced changes in apoptosis-related proteins, EMT-related proteins, and PI3K/AKT signalling

To further investigate whether IL-6 is involved in the regulation of apoptosis, EMT, and PI3K/AKT signalling by propranolol, Western blot analysis was performed in 786-O and Caki-2 cells from the control, propranolol treatment, IL-6 overexpression, and propranolol + IL-6 overexpression groups.

**FIGURE 6:**
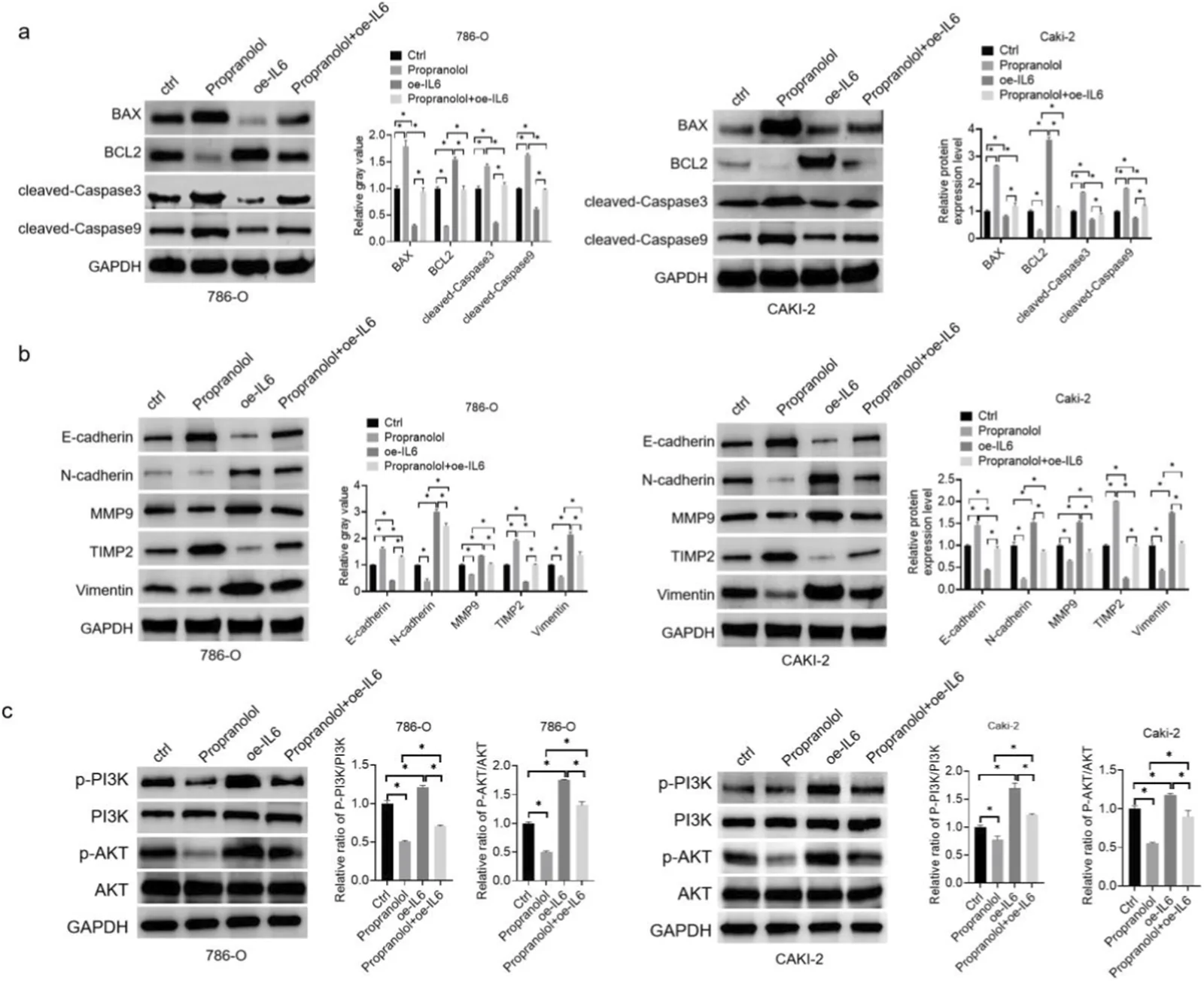
IL-6 overexpression partially reverses the effects of propranolol on apoptosis-related proteins, EMT-related proteins, and the PI3K/AKT signalling pathway in renal cancer cells. **(a)** Western blot analysis was performed to detect the expression levels of apoptosis-related proteins, including BAX, BCL-2, cleaved-Caspase-3, and cleaved-Caspase-9, in 786-O and Caki-2 cells from the control, propranolol, IL-6 overexpression, and propranolol + IL-6 overexpression groups. Grayscale quantitative analysis was also performed, with GAPDH used as the loading control. **(b)** Western blot analysis was performed to detect the expression levels of EMT- and invasion/metastasis-related proteins, including E-cadherin, N-cadherin, Vimentin, MMP9, and TIMP2, in 786-O and Caki-2 cells from the control, propranolol, IL-6 overexpression, and propranolol + IL-6 overexpression groups. Grayscale quantitative analysis was also performed, with GAPDH used as the loading control. **(c)** Western blot analysis was performed to detect the expression levels of PI3K/AKT signalling pathway-related proteins, including p-PI3K, PI3K, p-AKT, and AKT, in 786-O and Caki-2 cells from the control, propranolol, IL-6 overexpression, and propranolol + IL-6 overexpression groups. GAPDH was used as the loading control. Grayscale quantitative analysis of the p-PI3K/PI3K and p-AKT/AKT protein expression ratios in 786-O and Caki-2 cells. IL-6 overexpression partially restored propranolol-suppressed activation of the PI3K/AKT signalling pathway. Data are presented as the mean ± SD, n = 3 independent biological replicates. *P < 0.05.

For apoptosis-related proteins, propranolol treatment upregulated the expression of the pro-apoptotic proteins Bax, cleaved-Caspase-3, and cleaved-Caspase-9, while downregulating the expression of the anti-apoptotic protein Bcl-2. In contrast, IL-6 overexpression showed an opposite regulatory trend. Compared with the propranolol group, the propranolol + IL-6 overexpression group exhibited decreased expression of Bax, cleaved-Caspase-3, and cleaved-Caspase-9 and increased expression of Bcl-2, suggesting that IL-6 overexpression partially reversed propranolol-induced changes in apoptosis-related proteins (Figure 6a).

Western blot analysis of EMT- and invasion/metastasis-related proteins showed that propranolol upregulated the expression of the epithelial marker E-cadherin and the matrix metalloproteinase inhibitor TIMP2, while downregulating the expression of the mesenchymal markers N-cadherin and Vimentin, as well as the invasion-related protein MMP9. In contrast, IL-6 overexpression promoted an EMT-associated protein expression pattern. Compared with the propranolol group, the propranolol + IL-6 overexpression group showed decreased expression of E-cadherin and TIMP2 and increased expression of N-cadherin, Vimentin, and MMP9, suggesting that IL-6 overexpression partially reversed the regulatory effects of propranolol on EMT- and invasion/metastasis-related proteins (Figure 6b).

The PI3K/AKT signalling pathway was then examined to further clarify the potential mechanism underlying the rescue effect of IL-6. Western blot analysis showed that propranolol treatment reduced the phosphorylation levels of PI3K and AKT, as reflected by decreased p-PI3K/PI3K and p-AKT/AKT ratios. In contrast, IL-6 overexpression enhanced activation of the PI3K/AKT signalling pathway. Compared with the propranolol group, the propranolol + IL-6 overexpression group showed partial restoration of the p-PI3K/PI3K and p-AKT/AKT ratios (Figure 6c).

## 4 Discussion

In recent years, increasing evidence has shown that the nervous system is not merely a “bystander” within the tumour microenvironment, but an important component and active participant in tumour initiation and progression (Liu et al., 2025; Qiao et al., 2019). Complex bidirectional crosstalk exists between the nervous system and tumours, and this interaction can profoundly influence key biological processes, including tumour cell proliferation, immune evasion, angiogenesis, invasion, metastasis and therapeutic resistance. Accordingly, “neuro–tumour interactions” are emerging as an important research direction and potential therapeutic target in oncology.

Neurotransmitters, neurotrophic factors and neuropeptides can act on tumour cells, immune cells and vascular endothelial cells, thereby participating in the regulation of tumour progression (Liu et al., 2025; Qiao et al., 2019). Among these, β-blockers may exert multiple antitumour effects by blocking sympathetic β-adrenergic receptor (β-AR) signalling, including anti-proliferative, anti-metastatic and anti-angiogenic effects, improvement of the immunosuppressive state, and attenuation of tumour adaptation to stress-related microenvironments.

The present study demonstrated that propranolol exhibits antitumour activity in renal cancer cells. *In vitro* experiments showed that propranolol significantly inhibited the proliferative capacity of 786-O and Caki-2 cells in a concentration-dependent manner. In addition, propranolol suppressed renal cancer cell migration and invasion and promoted apoptosis, suggesting that it may interfere with the malignant biological behaviours of renal cancer cells at multiple levels. The propranolol concentrations (10–50 μM) used *in vitro* are higher than clinical plasma levels (0.1–1 μM). Higher concentrations may be required to elicit measurable biological effects in short-term static cell culture systems. Continuous treatment and intratumoral drug retention *in vivo* may achieve comparable inhibitory effects at lower systemic concentrations, and we recognize this pharmacokinetic limitation of our cellular experiments. Previous studies have indicated that β-adrenergic receptor signalling, particularly β2-AR signalling, may play an important role in the development and progression of clear cell renal cell carcinoma. Therefore, the antitumour effects of propranolol may be at least partly dependent on blockade of β-adrenergic receptor-related signalling pathways.

Previous studies in gastric cancer have shown that propranolol can induce G1-phase cell cycle arrest and promote apoptosis in MKN45 and NUGC3 cells, while downregulating the expression of MMP-2, MMP-9 and VEGF, thereby inhibiting cell migration, invasion and angiogenesis-related capacity (Koh et al., 2021). Consistent with these findings, the present study showed that propranolol treatment upregulated the expression of E-cadherin and TIMP2 in renal cancer cells, while downregulating N-cadherin, Vimentin and MMP9. E-cadherin is an important marker of the epithelial phenotype, whereas N-cadherin and Vimentin are closely associated with the mesenchymal phenotype and EMT progression. MMP9 promotes extracellular matrix degradation and enhances tumour cell invasion, whereas TIMP2 inhibits matrix metalloproteinase activity. These findings suggest that propranolol may attenuate the migratory and invasive capacities of renal cancer cells by suppressing EMT progression and extracellular matrix remodelling.

The antitumour mechanisms of propranolol may involve multi-pathway and multi-level regulation, including tumour cell proliferative signalling, inflammatory responses, angiogenesis, mesenchymal phenotypic transition and remodelling of the tumour microenvironment. Previous studies have shown that propranolol can inhibit activation of the HIF-2α/VEGF pathway and AKT/MAPK signalling, improve CD8^+^ T-cell infiltration within the tumour immune microenvironment, suppress tumour cell proliferation and reduce Ki-67 expression (Hu et al., 2021; Shepard et al., 2018; Albiñana et al., 2015; Yang et al., 2023). In addition, the combination of propranolol with anti-CTLA-4 therapy has been reported to further enhance antitumour efficacy (Fjæstad et al., 2022). In the present study, the nude mouse xenograft experiments further demonstrated that propranolol significantly delayed tumour growth and reduced the final tumour volume and weight. Immunohistochemical analysis also showed decreased Ki-67 expression in tumour tissues after propranolol treatment, consistent with reduced proliferative activity within the tumour tissues. These findings further support the potential antitumour effect of propranolol in renal cancer. However, because the xenograft experiments were performed in nude mice, the potential contribution of host immune-mediated antitumour effects cannot be excluded.

Among the key signalling pathways involved in RCC development and progression, the PI3K/AKT pathway has attracted considerable attention because of its role in regulating tumour cell proliferation, survival, migration, invasion, angiogenesis, metabolic reprogramming and therapeutic resistance (Ding et al., 2022; Hwang et al., 2024; Wang et al., 2021). Accordingly, the PI3K/AKT pathway is regarded as an important molecular regulatory axis in RCC progression and a potential therapeutic target.

In the present study, RNA-seq analysis showed marked transcriptomic remodelling in renal cancer cells after propranolol treatment, with differentially expressed genes significantly enriched in the PI3K/AKT pathway, extracellular matrix remodelling, cell adhesion, inflammatory cytokine networks and metabolism-related pathways. Further Western blot analysis showed that propranolol reduced the p-PI3K/PI3K and p-AKT/AKT ratios, indicating inhibition of PI3K/AKT pathway activation. Together with the functional assay and rescue experiment results, these findings suggest that suppression of PI3K/AKT signalling contributes, at least in part, to the inhibitory effects of propranolol on the malignant biological behaviours of renal cancer cells. Previous studies have reported ADRB2 expression in RCC tissues and RCC cell lines, including 786-O and Caki-2. We recognize that direct detection of cellular ADRB2 protein/mRNA levels is absent in the present study, and related expression validation experiments will be performed in our subsequent research to further consolidate this mechanistic axis.

Notably, the expression pattern and prognostic significance of ADRB2/β2-AR are highly tumour type-specific. Previous studies have shown that high β2-AR expression is associated with tumour progression and poor prognosis in gastric cancer, whereas in ccRCC, some cohort studies have suggested that low ADRB2 expression may be associated with advanced stage, higher grade and poorer overall survival (Ha et al., 2019; Koh et al., 2021). This discrepancy indicates that the biological function of ADRB2/β2-AR may be influenced by tumour tissue origin, differentiation status, the tumour microenvironment and downstream signalling networks. Previous studies have suggested that ADRB2 blockade may attenuate inflammatory signalling in ccRCC models, whereas IL6 can activate the PI3K/AKT signalling pathway and promote ccRCC progression (Lu et al., 2024). Consistent with these findings, our results showed that propranolol treatment markedly reduced IL6 expression and suppressed PI3K/AKT pathway activation. Functional rescue experiments further supported the involvement of this pathway. The partial rescue effect observed upon IL-6 overexpression indicates that IL-6/PI3K/AKT is not the sole downstream signalling axis. As mentioned in the Introduction, other tumour-relevant factors such as HIF-2α may also participate in mediating the anti-cancer function of propranolol, which deserves further investigation in future research. These findings suggest that the ADRB2-related IL6/PI3K/AKT axis may partially contribute to the antitumour effects of propranolol. Future studies integrating clinical cohorts, β-blocker medication history and molecular subtyping are needed to further clarify the biological significance of ADRB2/β2-AR signalling in RCC.

In addition to the IL-6/PI3K/AKT axis, RNA-seq analysis revealed significant enrichment of several pathways associated with tumour–microenvironment interactions and metastatic progression, including ECM–receptor interaction, focal adhesion, and cytokine–cytokine receptor interaction, where ECM–receptor interaction and focal adhesion signalling regulate the adhesion of tumour cells to the extracellular matrix and participate in cytoskeletal remodelling, cell migration, invasion, and metastasis (Majo et al., 2020), while cytokine–cytokine receptor interaction pathways may mediate communication among tumour cells and between tumour cells and components of the tumour microenvironment through cytokines such as IL-6, thereby influencing tumour growth, cell survival, inflammatory responses, and therapeutic responses (Chen et al., 2018). In addition, the enrichment of MAPK, HIF-1, apoptosis-related, and metabolic pathways suggests that propranolol may also affect stress adaptation, cell survival, programmed cell death, and metabolic reprogramming in renal cancer cells, with MAPK and HIF-1 signalling in particular being closely associated with tumour-cell proliferation, adaptation to hypoxia, and angiogenesis (Zhang et al., 2010; Turner et al., 2002). Collectively, these findings suggest that propranolol may exert its antitumour effects through the coordinated modulation of multiple tumour-related signalling pathways rather than through a single downstream pathway. The functional rescue experiments performed in the present study support the IL-6/PI3K/AKT axis as an important mechanistic component, but not the only pathway involved. Further mechanistic studies are required to identify the key molecules within the other enriched pathways and clarify their functional relationships with β-adrenergic signalling.

This study has several limitations. First, although RNA-seq identified multiple tumour-related pathways potentially affected by propranolol, their functional contributions were not systematically validated in the present study. Likewise, changes in immune-cell infiltration, inflammatory cytokine networks, and microenvironmental remodelling require further investigation. Second, the 786-O xenograft experiments were performed in nude mice. Although this model supports the evaluation of tumour cell-autonomous effects, nude mice retain residual immune components, including NK cells. Therefore, host-mediated mechanisms may also contribute to the antitumour effects of propranolol *in vivo*. Further studies using an immunocompetent orthogonal model, together with immune-cell profiling or NK-cell depletion experiments, are needed to distinguish direct tumour cell effects from host-mediated responses. Third, the potential value of propranolol in combination with targeted therapy or immunotherapy was not evaluated. Fourth, given the tumour type-specific biological significance of ADRB2/β2-AR, future studies integrating clinical cohorts, β-blocker medication history and ADRB2 expression levels are needed to further clarify its prognostic significance and therapeutic value in RCC. Fifth, the *in vivo* validation was mainly based on the measurement of total mRNA levels of PI3K, AKT, IL-6, and other tumour-related genes. Because PI3K/AKT pathway activity is primarily regulated by protein phosphorylation, changes in PI3K and AKT mRNA expression cannot be directly interpreted as evidence of altered pathway activation. Therefore, the tumour qPCR findings should be regarded as transcriptional support consistent with the proposed mechanism, rather than direct evidence of PI3K/AKT pathway activity. Further studies assessing phosphorylated PI3K and AKT levels in tumour tissues by western blotting or immunohistochemistry are required to confirm the *in vivo* activation status of this pathway. Finally, only a single concentration of propranolol (20 μM) was used in the IL-6 overexpression rescue experiments, which may not fully characterize the dose dependence of the interaction between propranolol and IL-6 signalling. Future studies using multiple propranolol concentrations are needed to determine whether the IL-6-mediated rescue effect is dose-dependent.

In conclusion, the present study demonstrates that propranolol significantly inhibits renal cancer cell proliferation, migration and invasion, promotes apoptosis, and suppresses tumour growth in a nude mouse xenograft model. Mechanistically, propranolol remodelled the transcriptome of ccRCC cells, downregulated IL-6, and inhibited PI3K/AKT pathway activation and regulated EMT-related protein expression while IL-6 overexpression partially reversed these anti-tumour phenotypes. As a cheap, clinically safe repurposed oral drug, propranolol holds promising translational potential for adjuvant treatment of clear cell renal cell carcinoma. Nevertheless, this work is limited by the gap between *in vitro* drug concentrations and clinical plasma exposure, and further pharmacokinetic and combined therapeutic studies are required before clinical application.

## Data Availability

The RNA-sequencing data generated in this study have been deposited in the Genome Sequence Archive for Human (GSA-Human), National Genomics Data Center, China National Center for Bioinformation, under accession number HRA019961. The dataset is currently maintained under confidential access and will be publicly available upon its scheduled release.
