# Supplementary material for: Propranolol attenuates the malignant biological behaviors of renal cancer cells partly through modulation of the IL-6/PI3K/AKT signaling axis
Source: Front Pharmacol. 2026 Sep 11;17:1884603. doi: 10.3389/fphar.2026.1884603 (PMC13612918; doi:10.3389/fphar.2026.1884603)
Supplement: Supplementary file 1 [file DataSheet1.pdf]

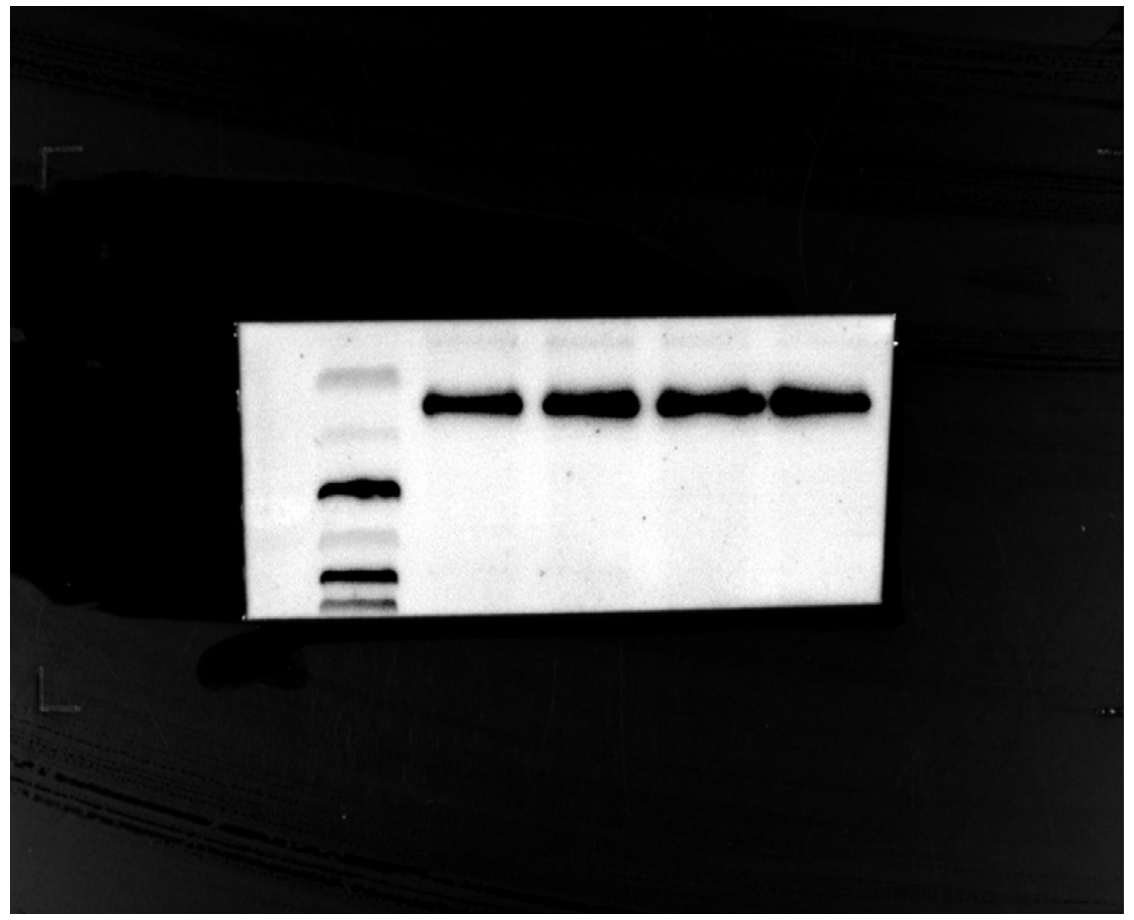

PI3K-merge-786-O

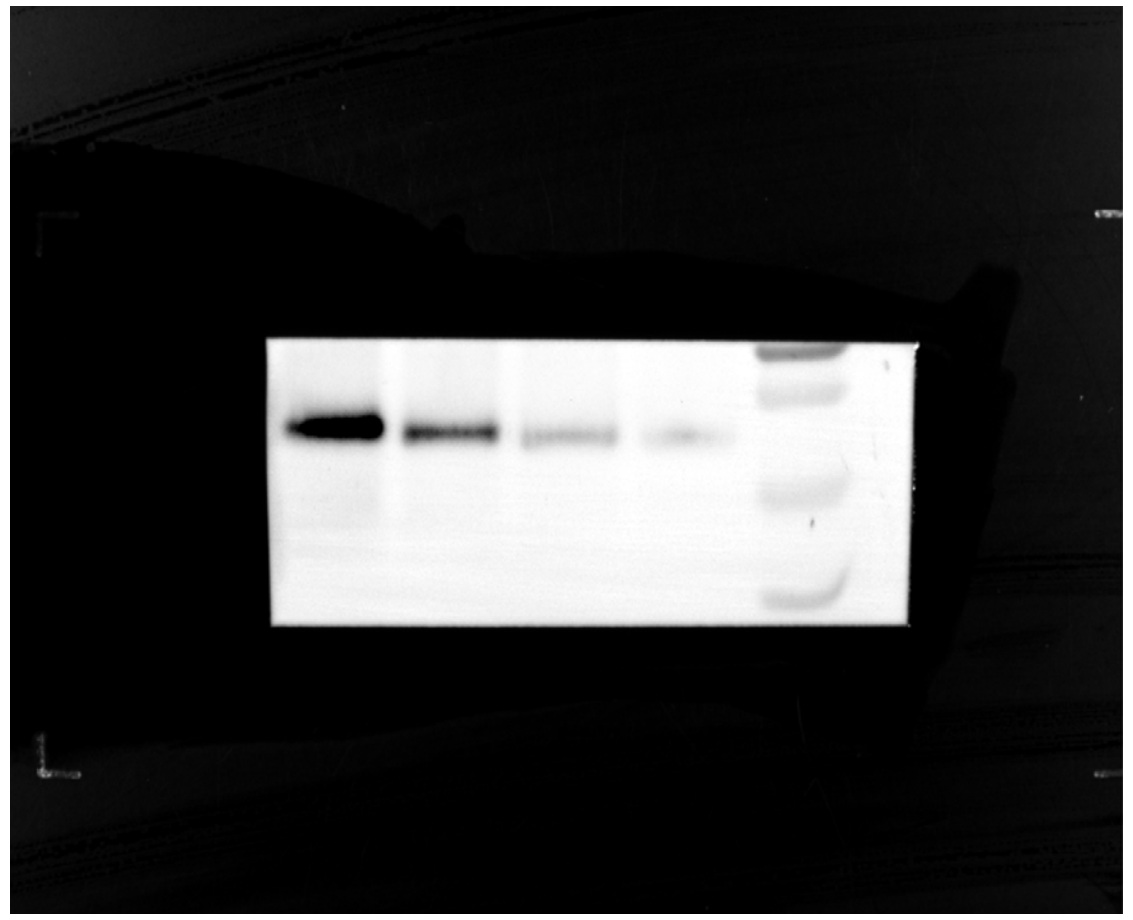

p-AKT-merge-786-O

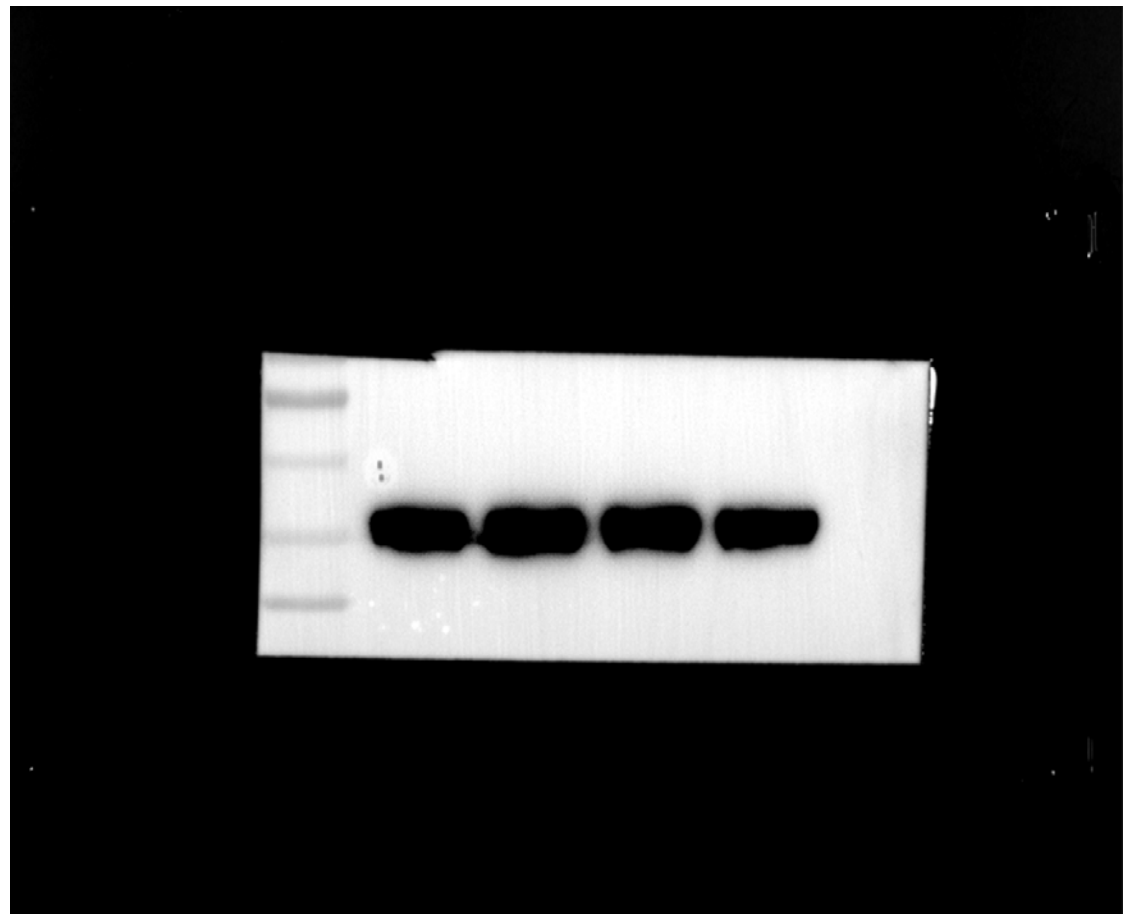

β-actin-merge-786-o

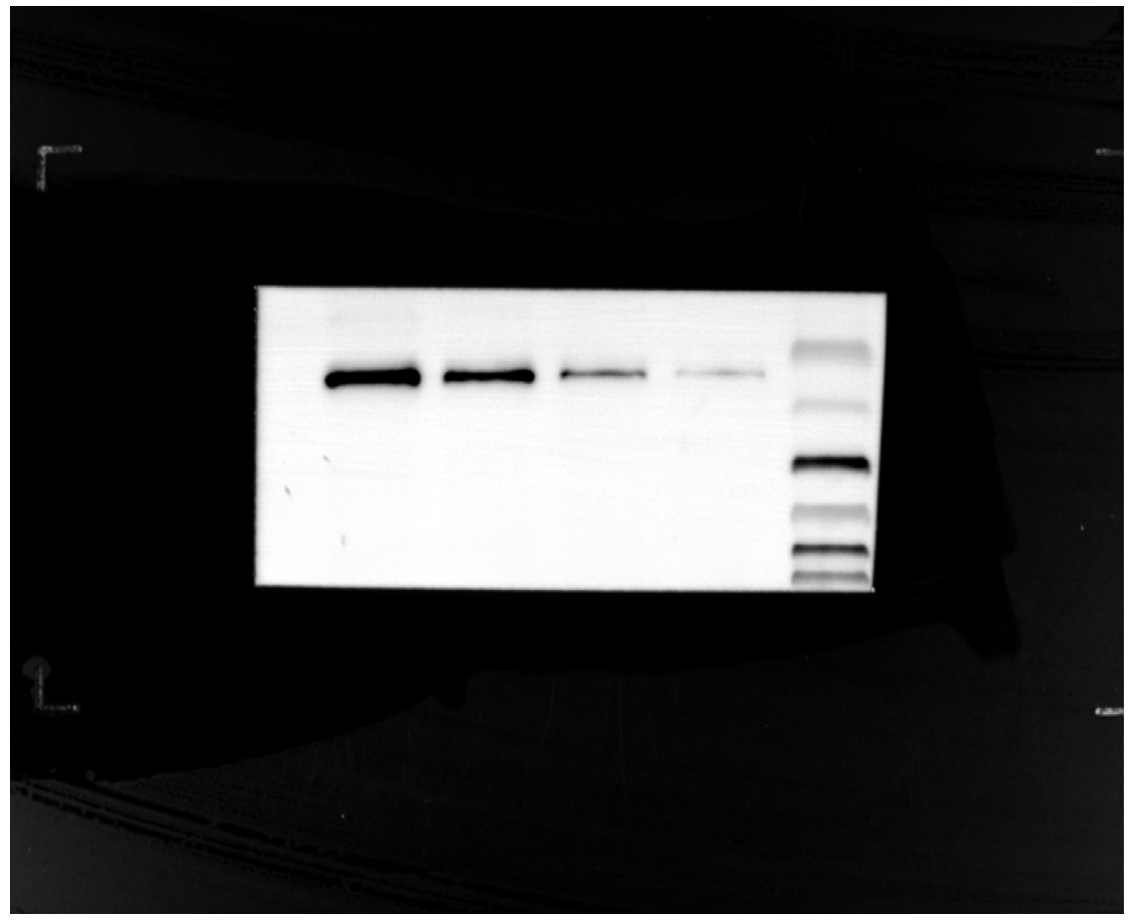

p-PI3K-merge-786-O

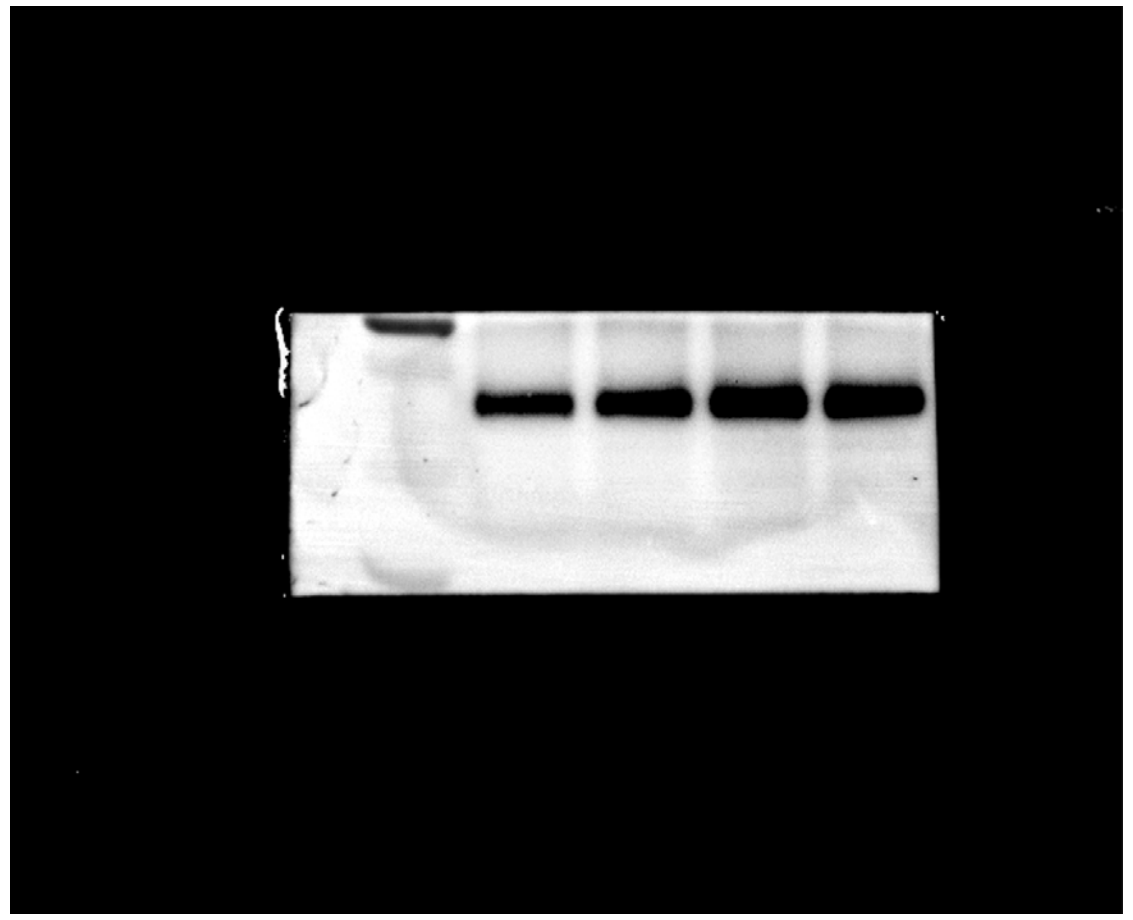

AKT-merge-786-O

Supplementary Figure S1. Full uncropped Western blot images for PI3K/AKT signalling proteins in 786-O cells.

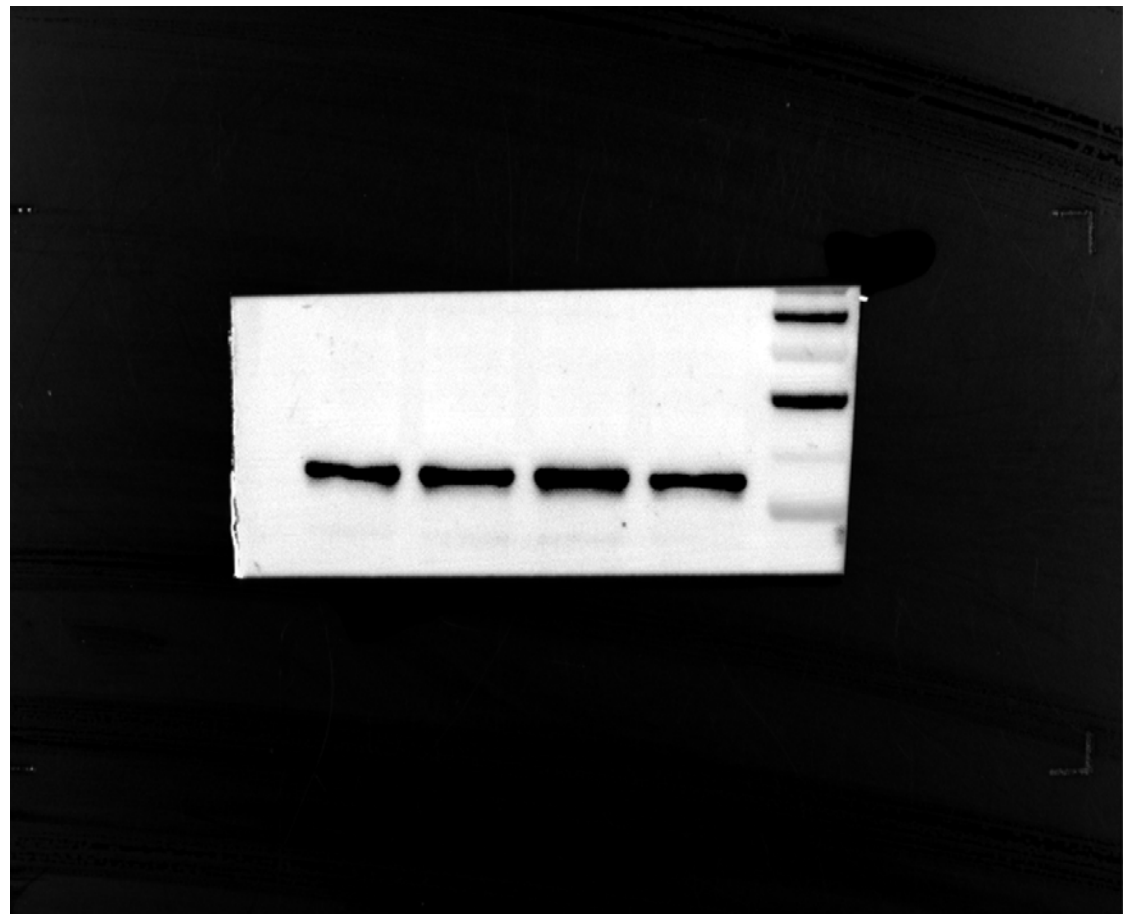

PI3K-merge-caki2

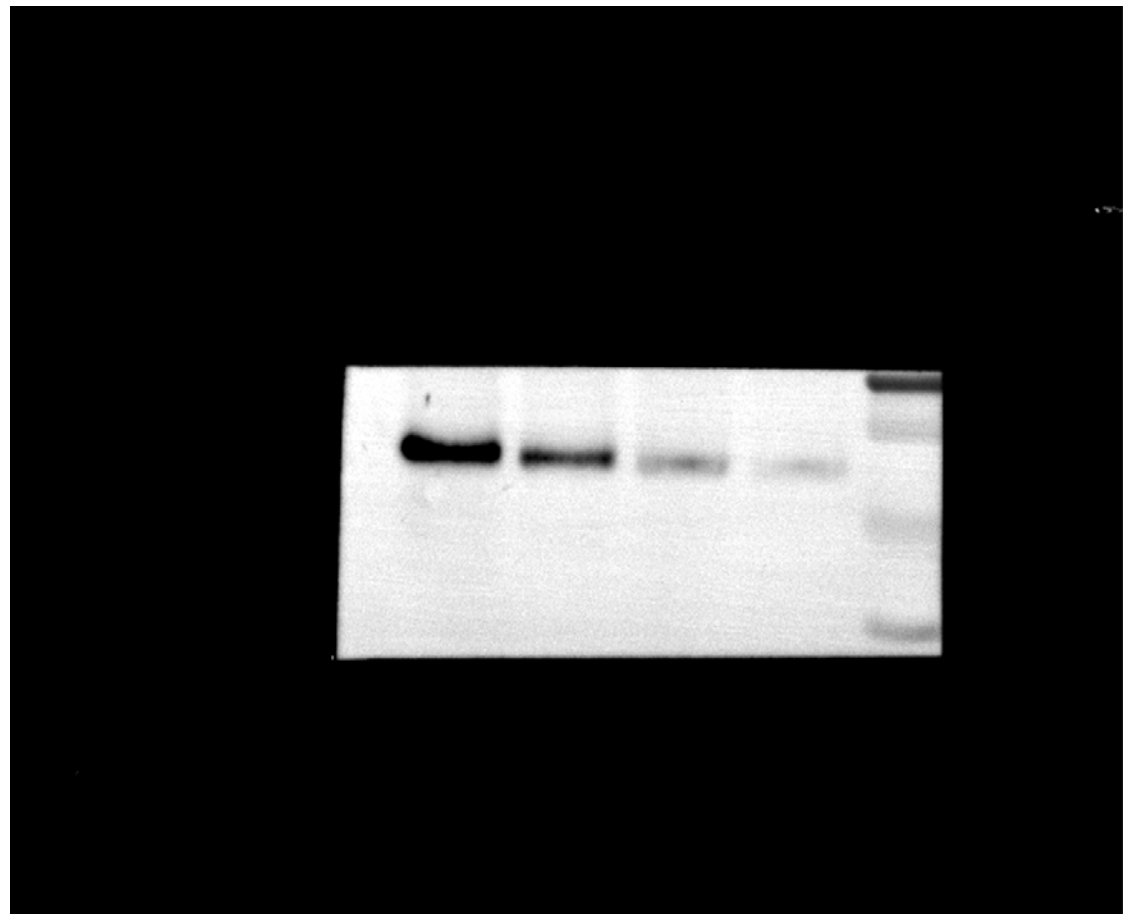

p-AKT-merge-caki2

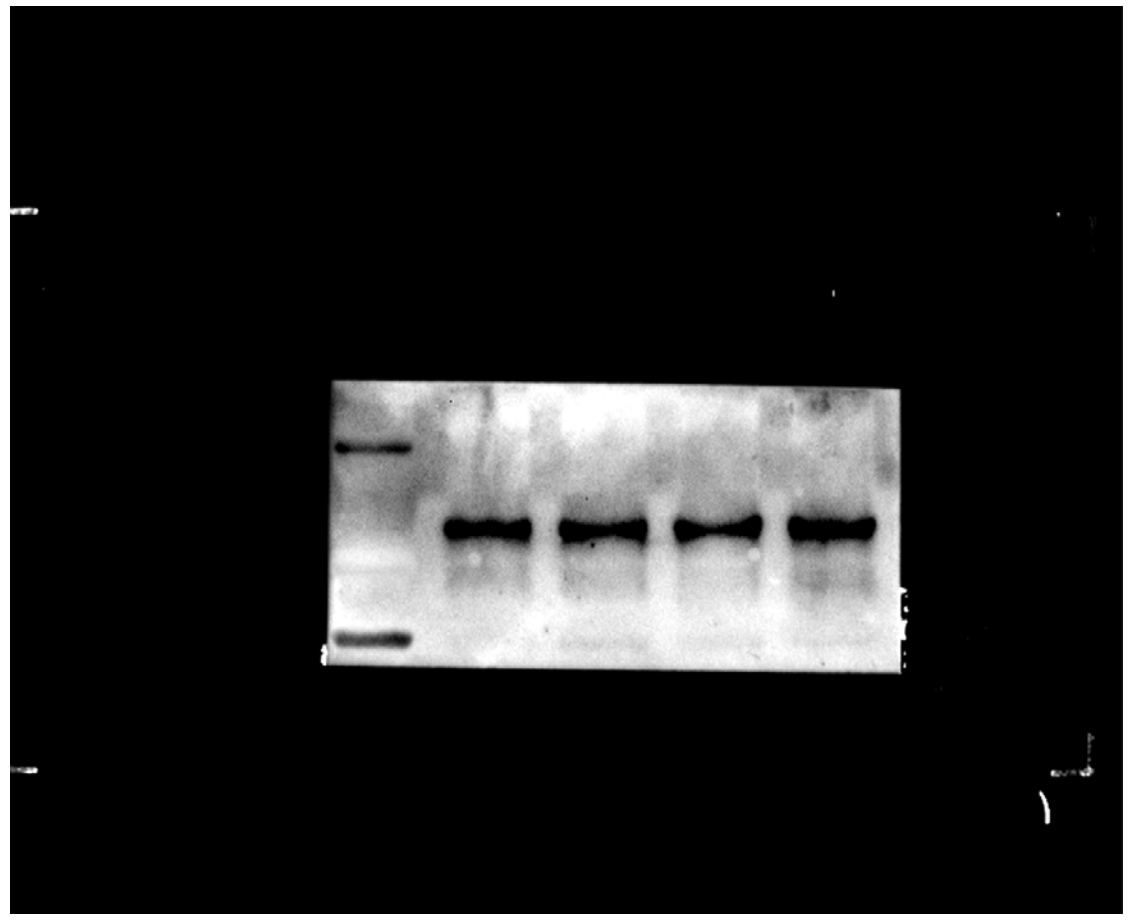

β-actin-merge-caki2

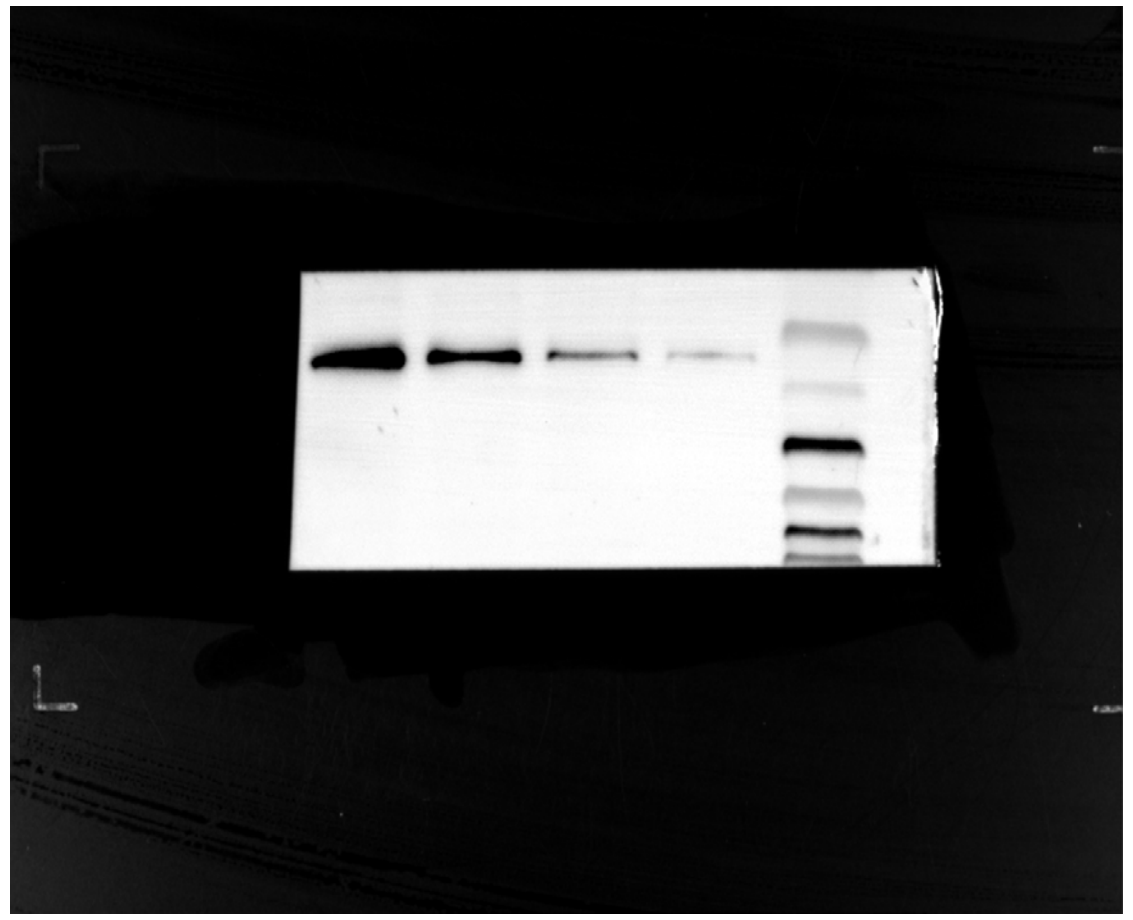

p-PI3K-merge-caki2

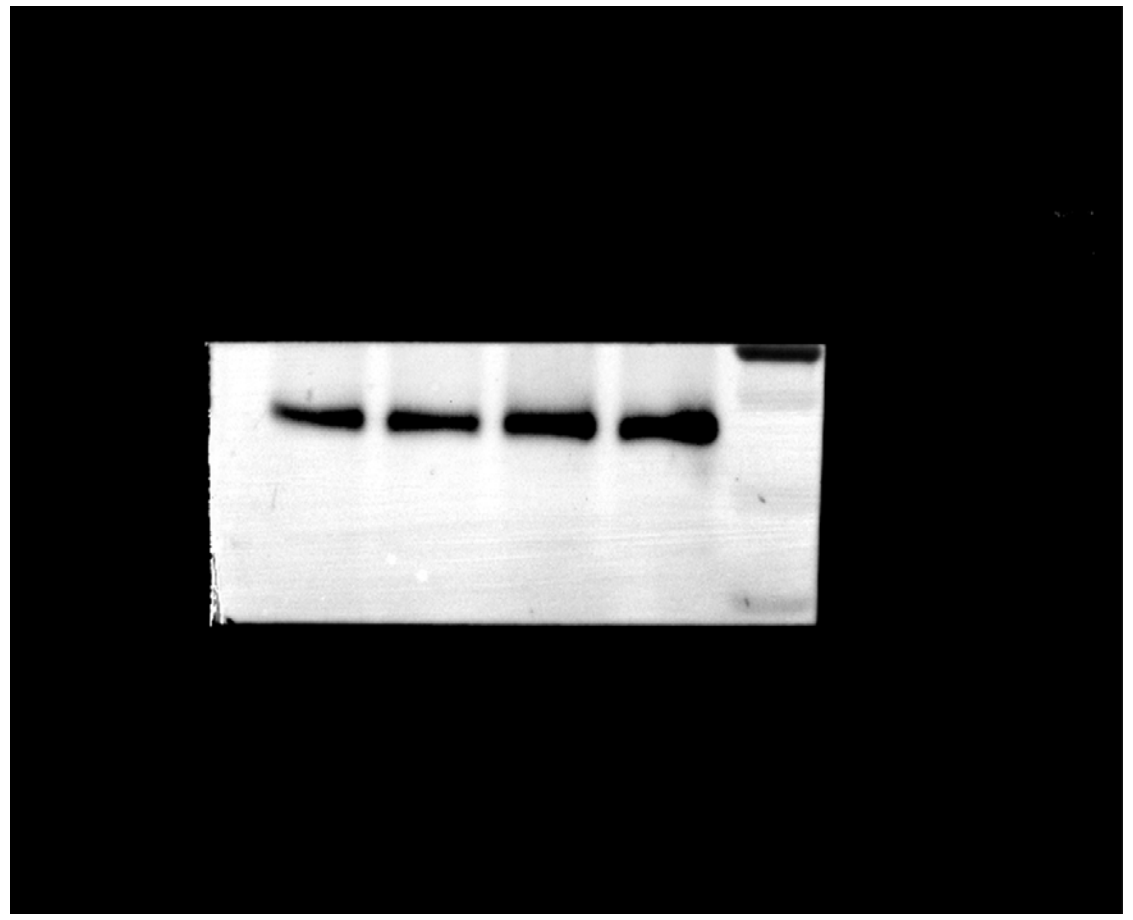

AKT-merge-caki2

Supplementary Figure S2. Full uncropped Western blot images for PI3K/AKT signalling proteins in Caki-2 cells.

Supplementary Figure S3. Full uncropped Western blot images for apoptosis-related proteins in 786-O cells.

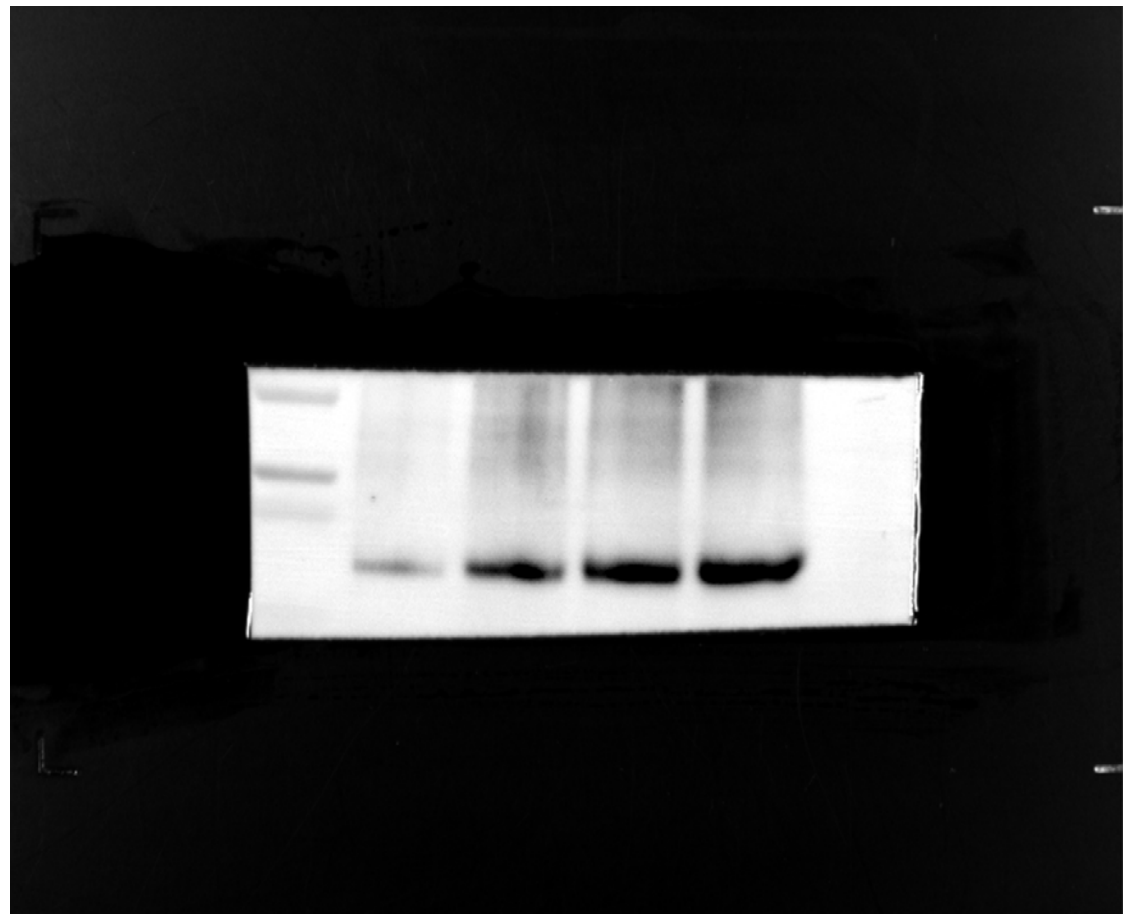

BAX-1-786-o

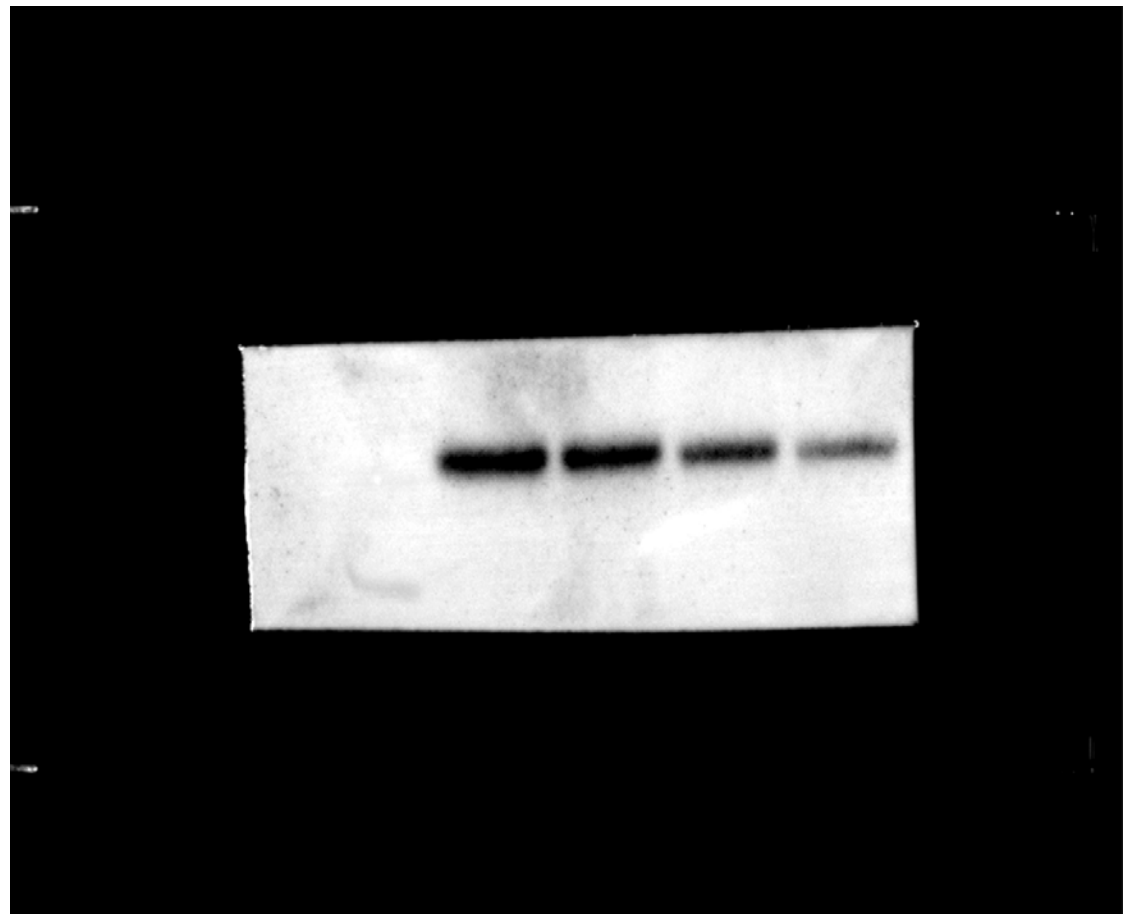

BCL2-1-786-O

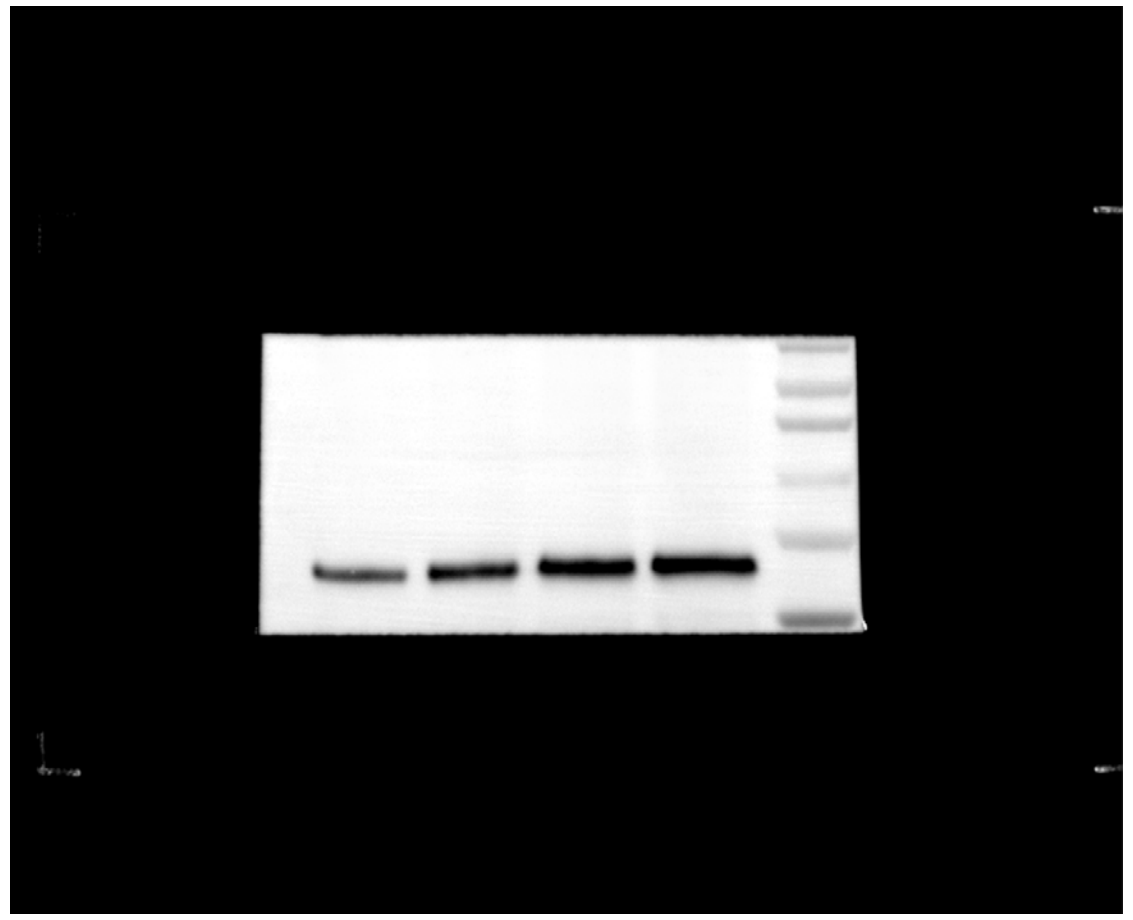

caspase3-1-786-o

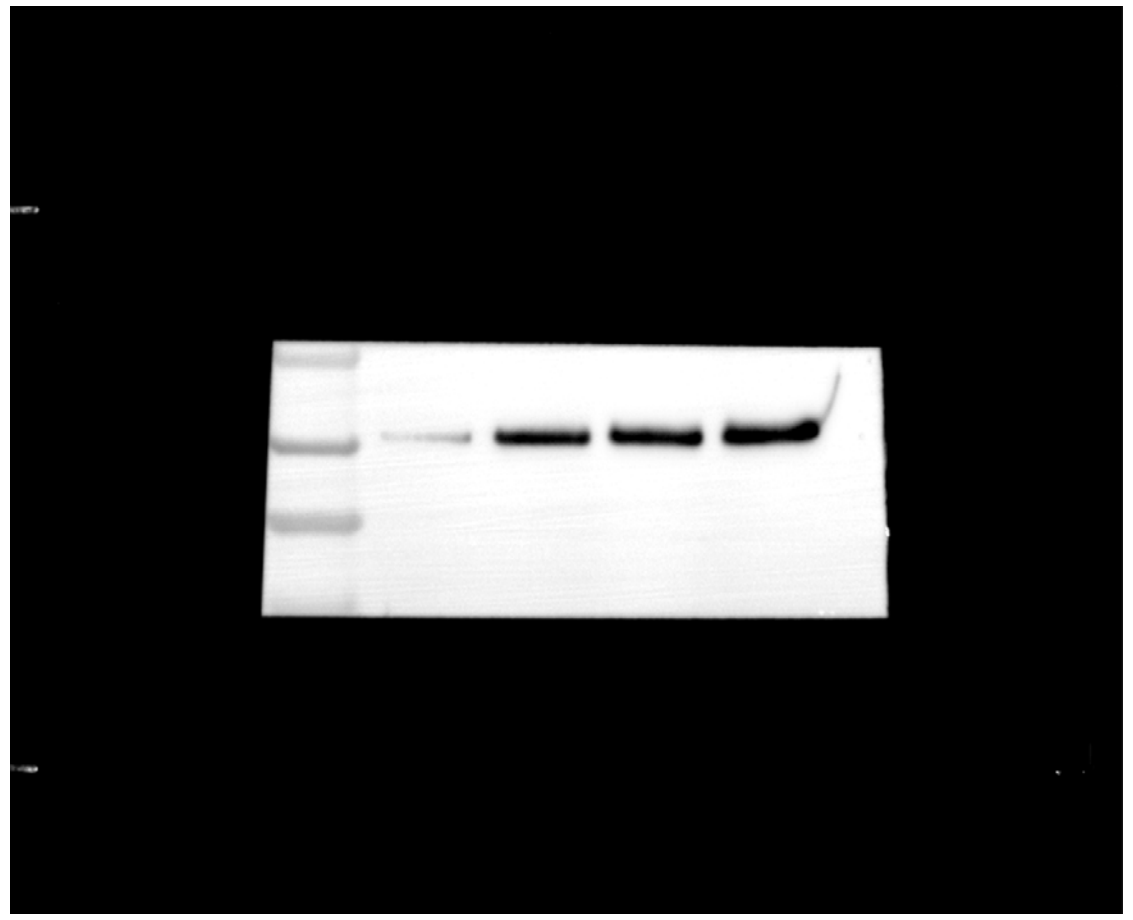

caspase9-1-786-o

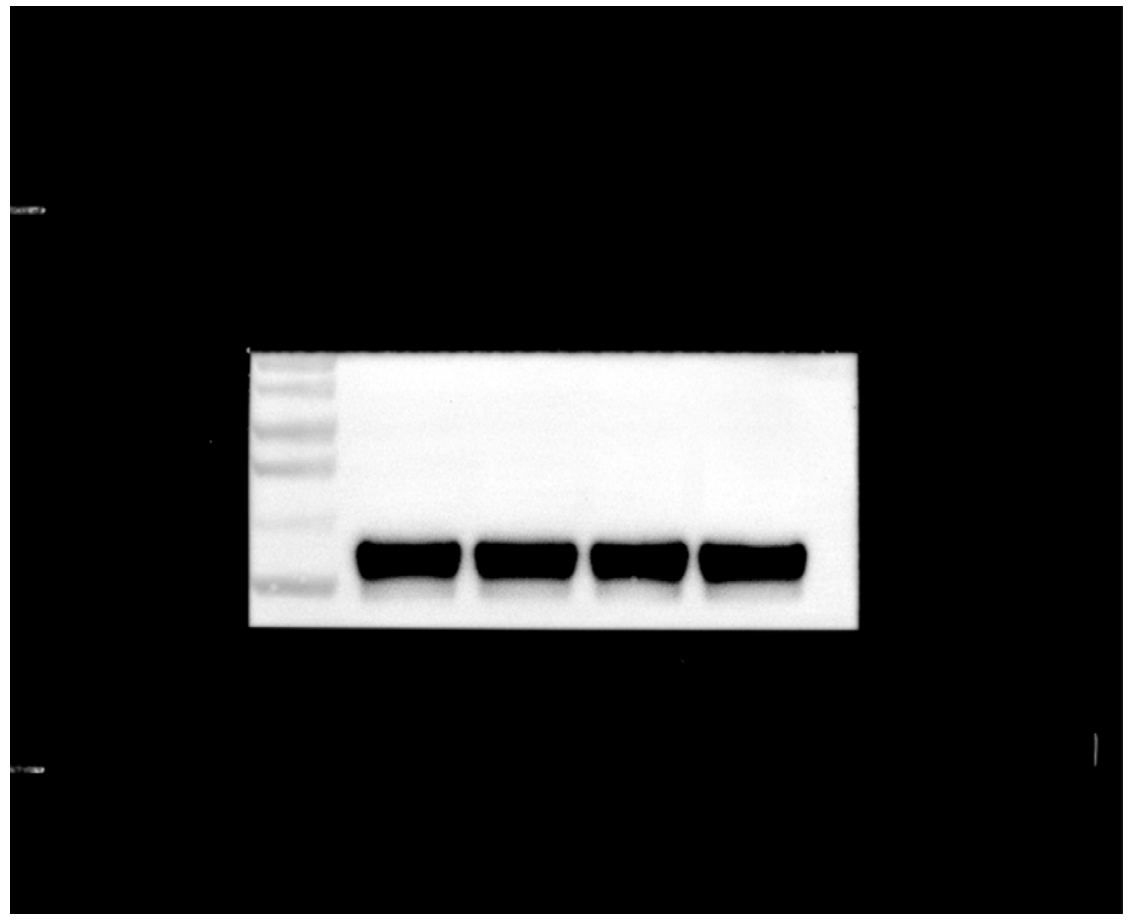

β-actin-1-786-o

Supplementary Figure S4. Full uncropped Western blot images for apoptosis-related proteins in Caki-2 cells.

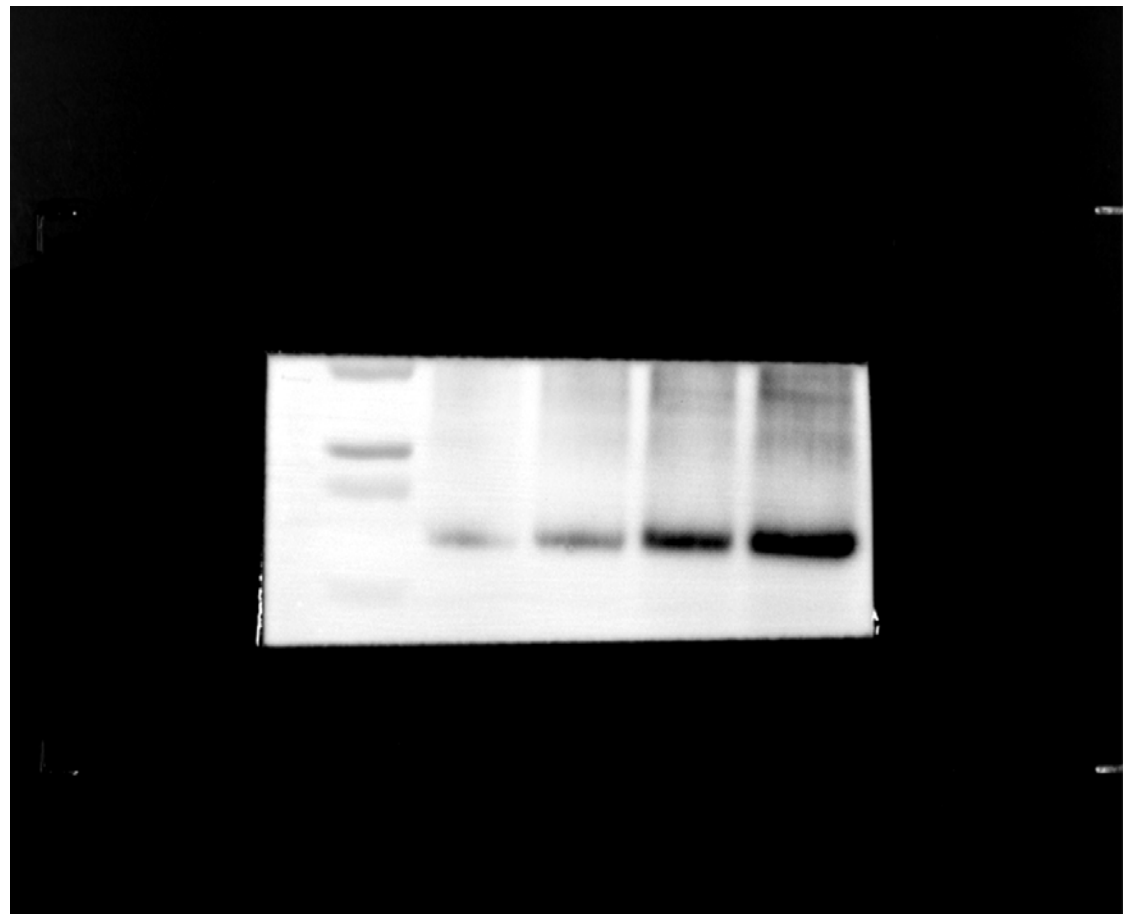

BAX-1-caki2

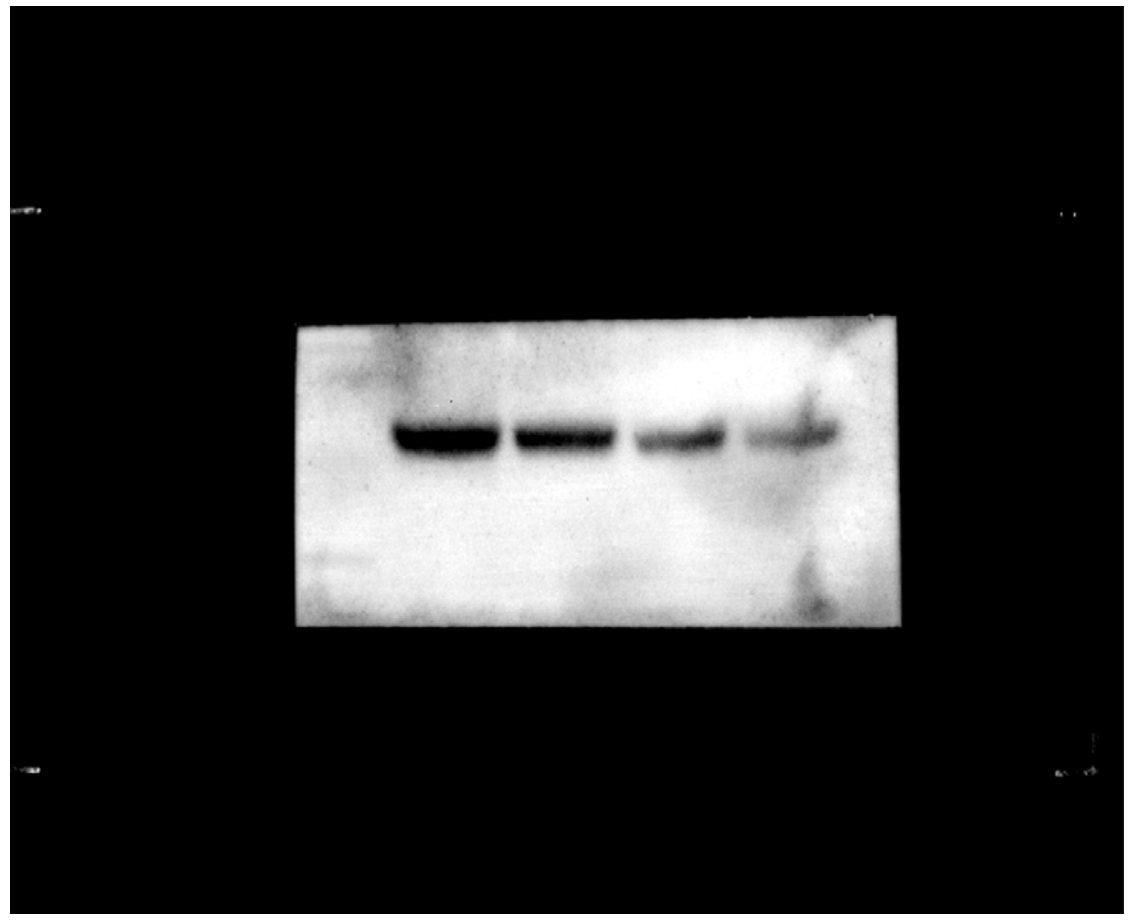

BCL2-1-caki2

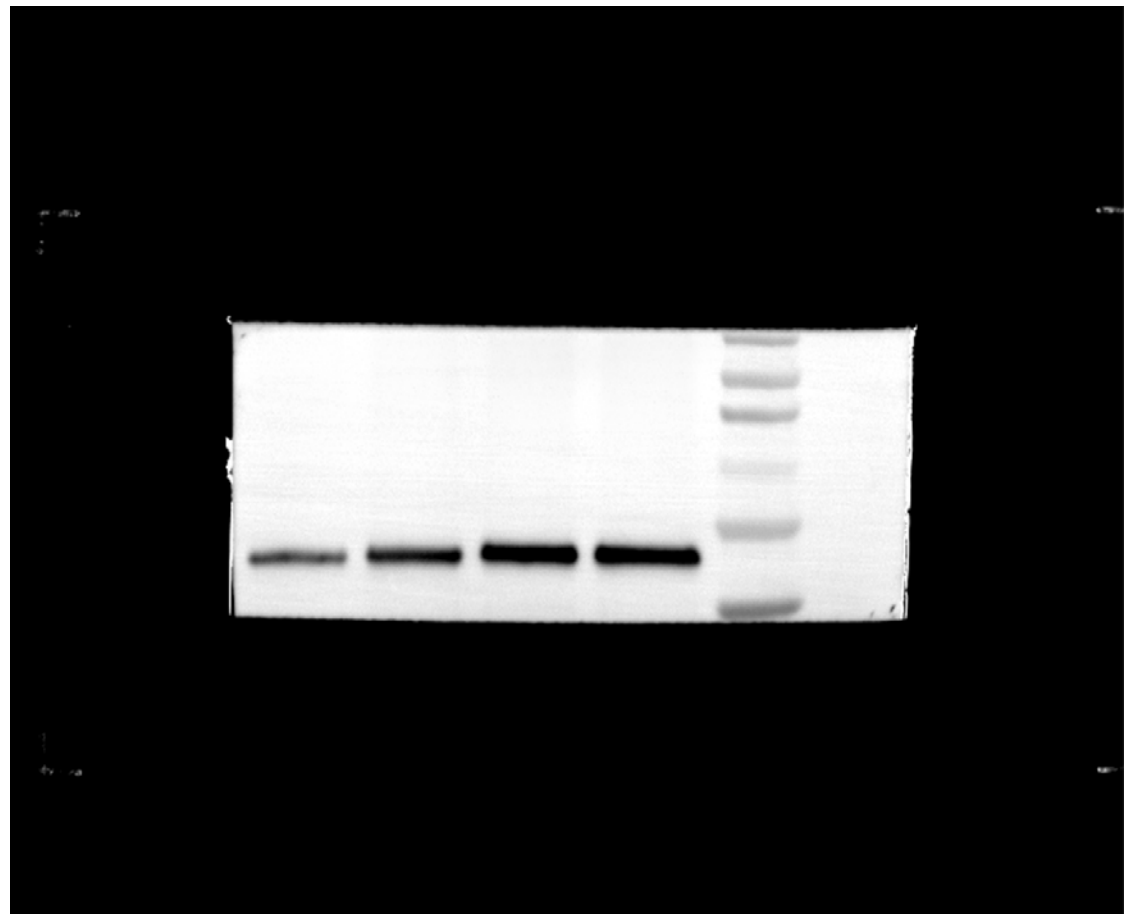

caspase3-1-caki2

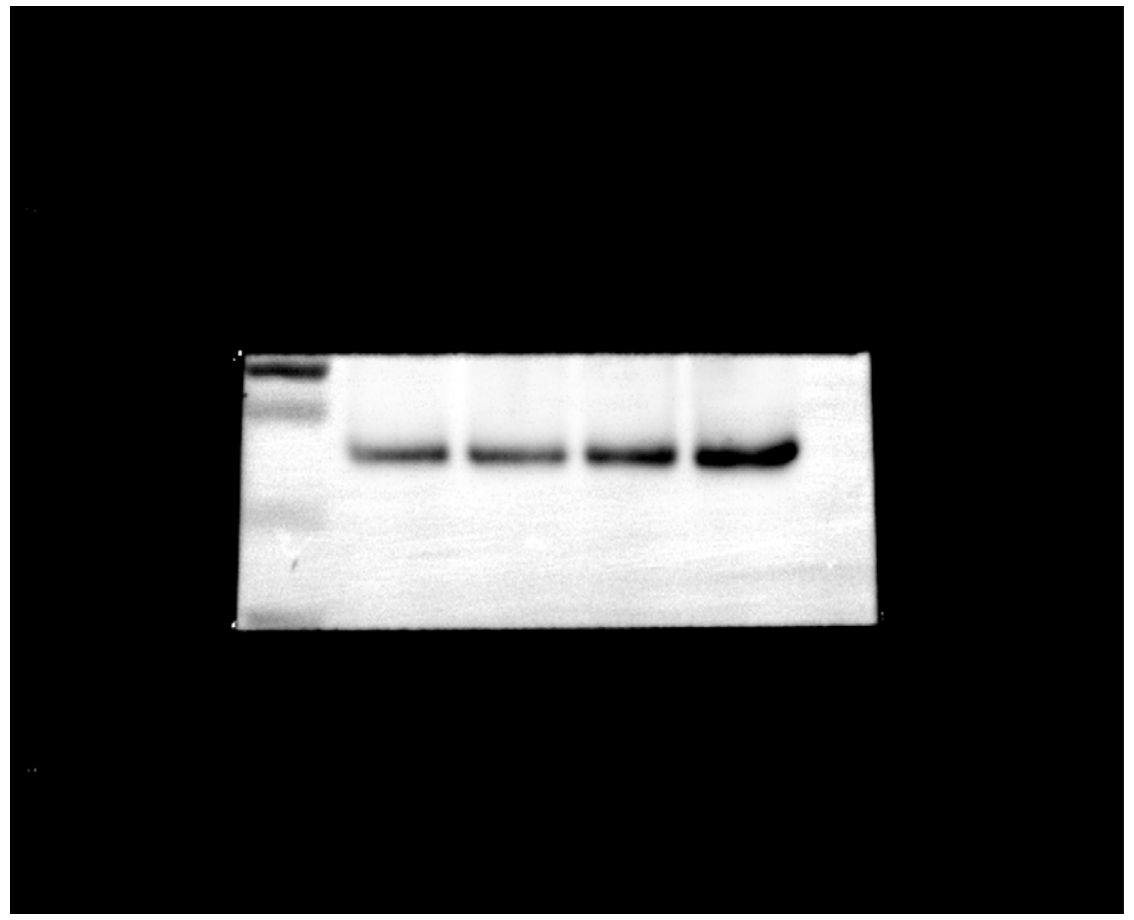

caspase9-1-caki2

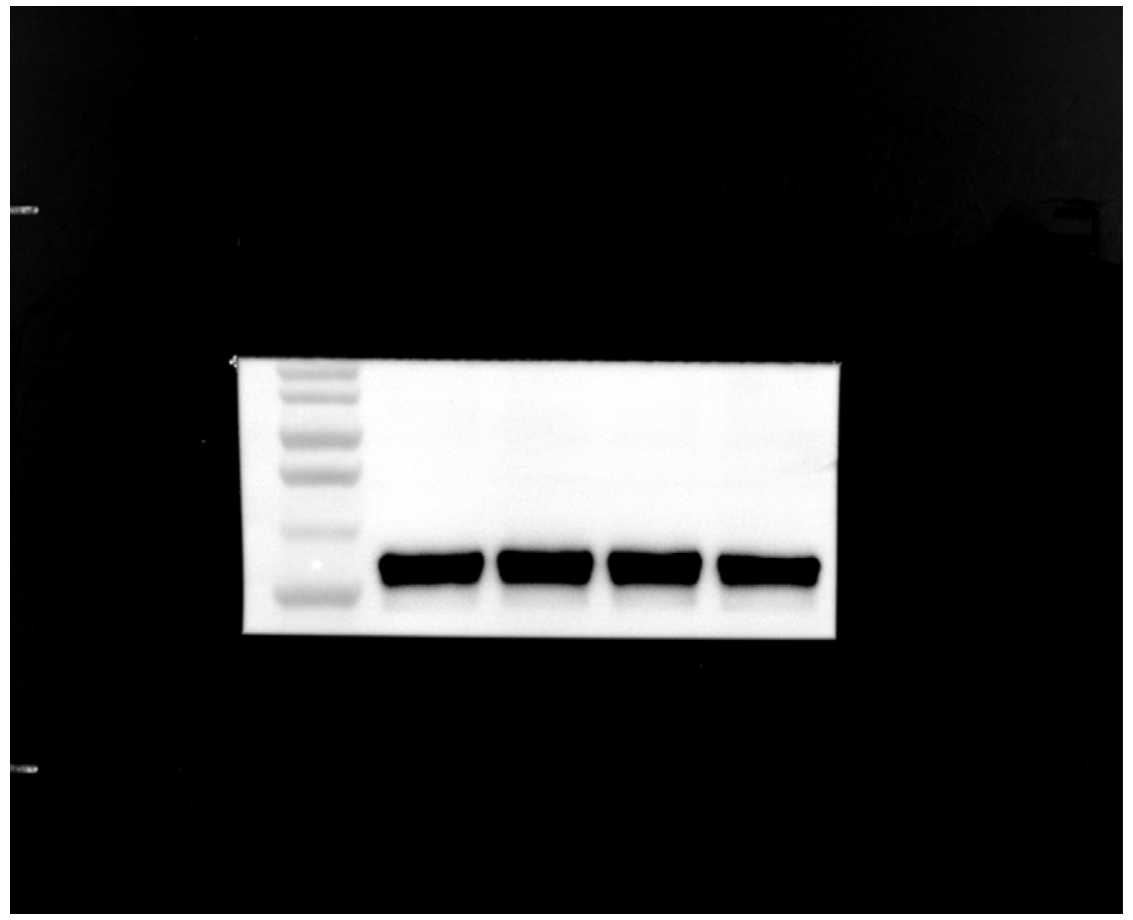

β-actin-1-caki2

Supplementary Figure S5. Full uncropped Western blot images for EMT- and invasion-related proteins in 786-O cells.

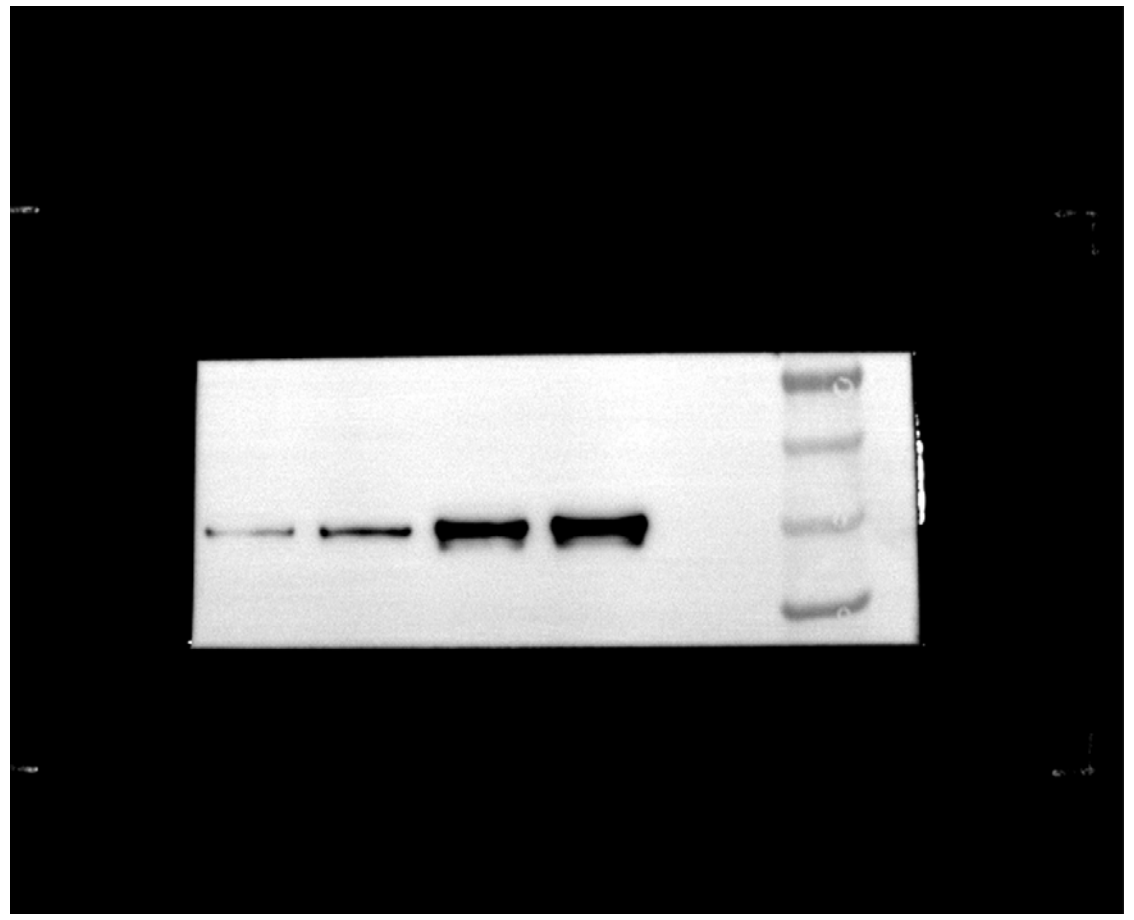

E-cadherin-1-786-o

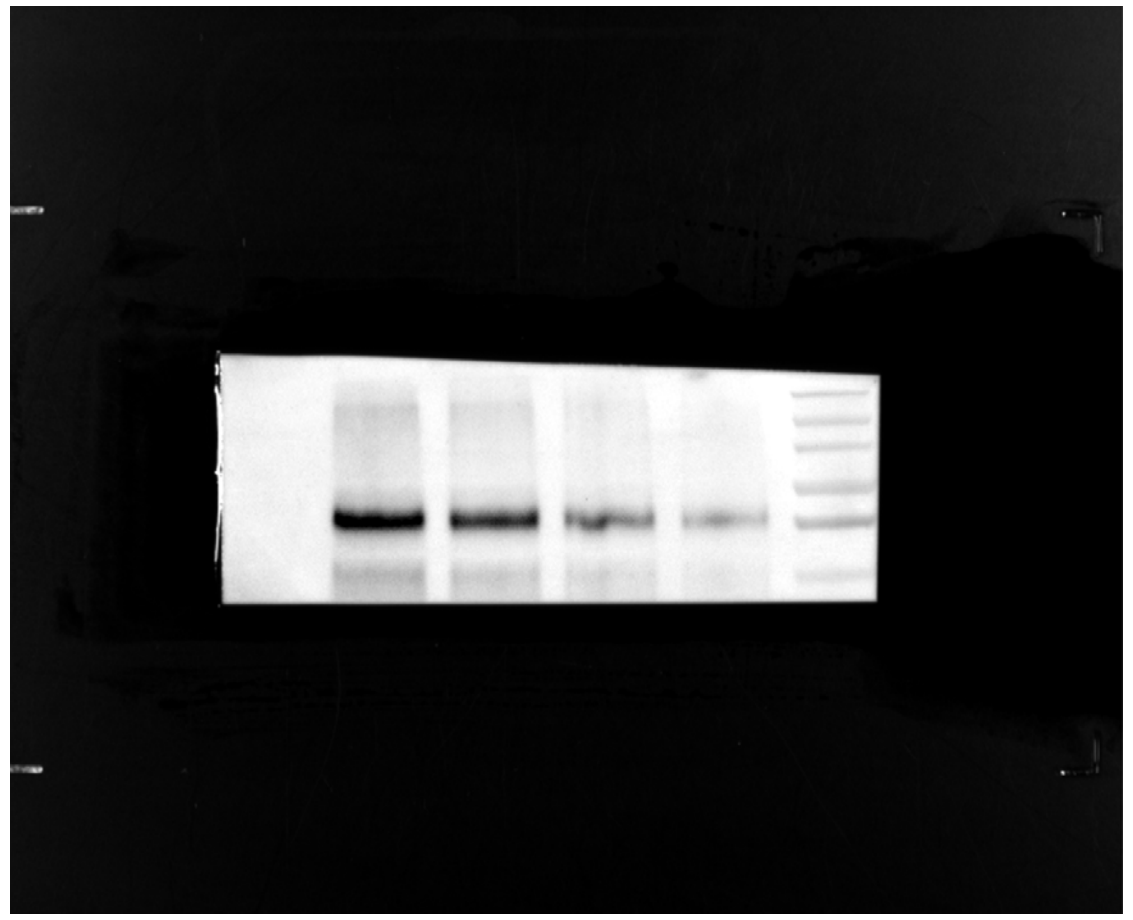

MMP9-1-786-o

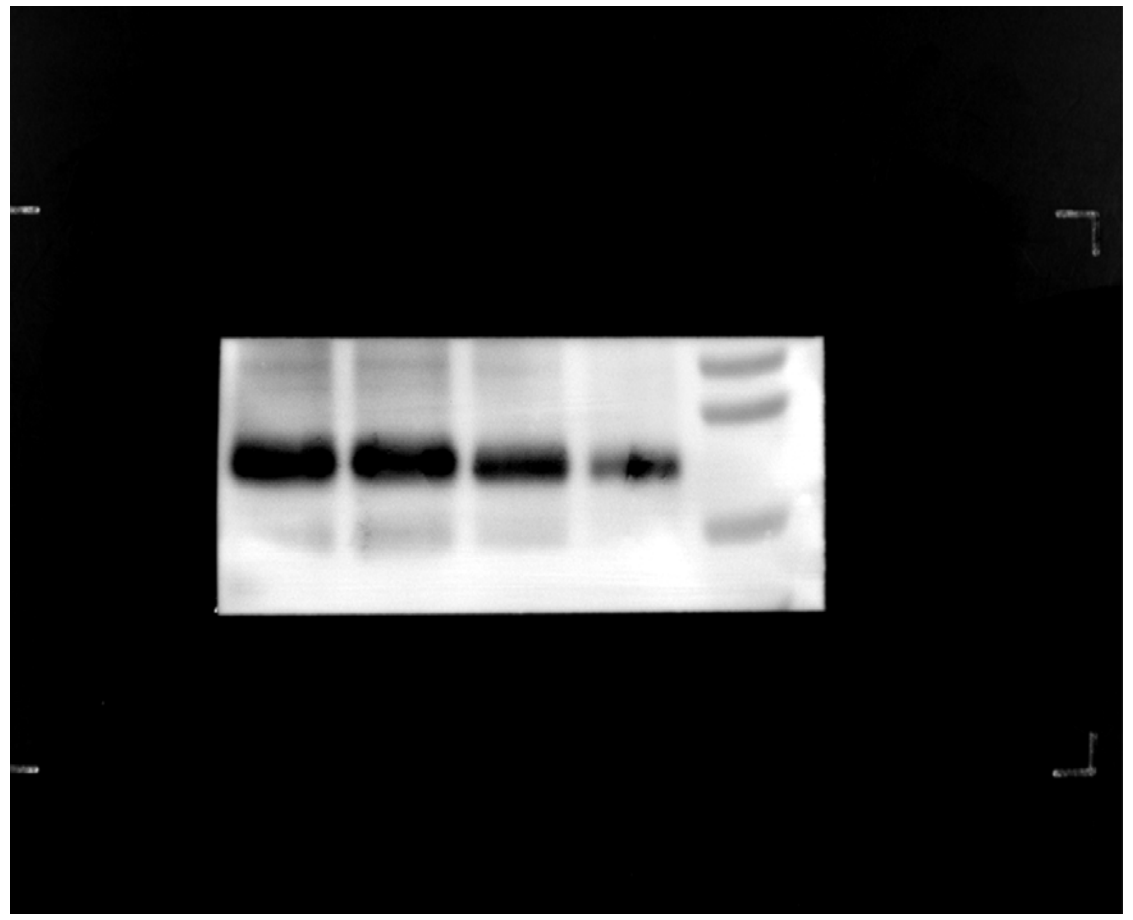

N-cadherin-1-786-o

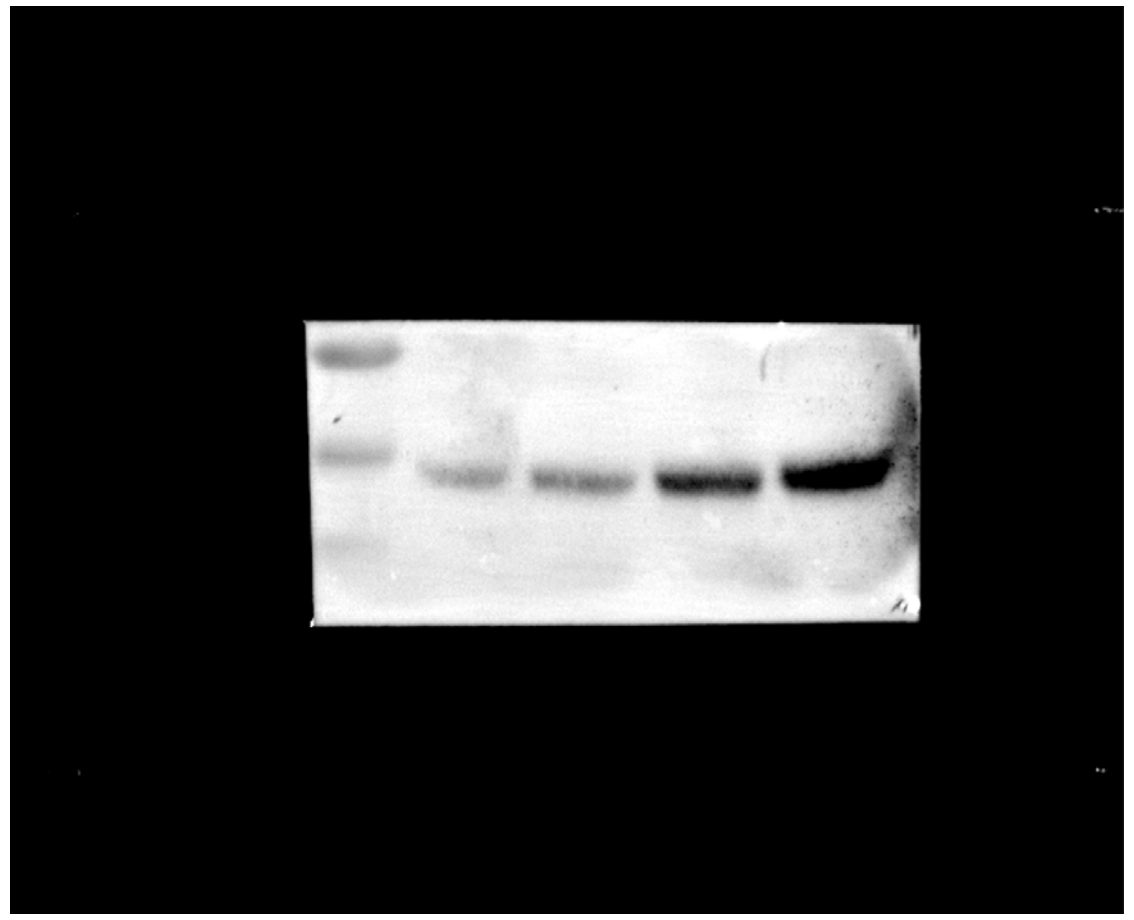

TIMP2-1-786-o

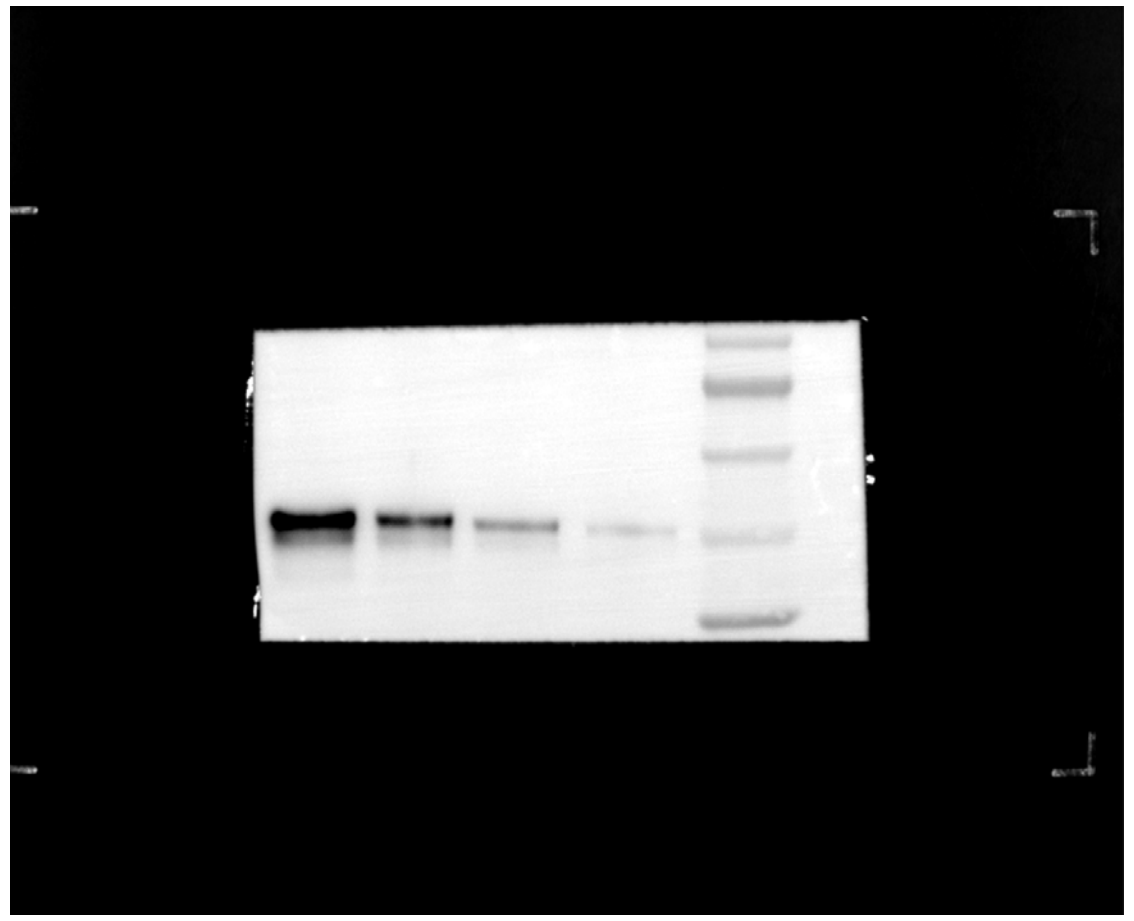

Vimentin-1-786-o

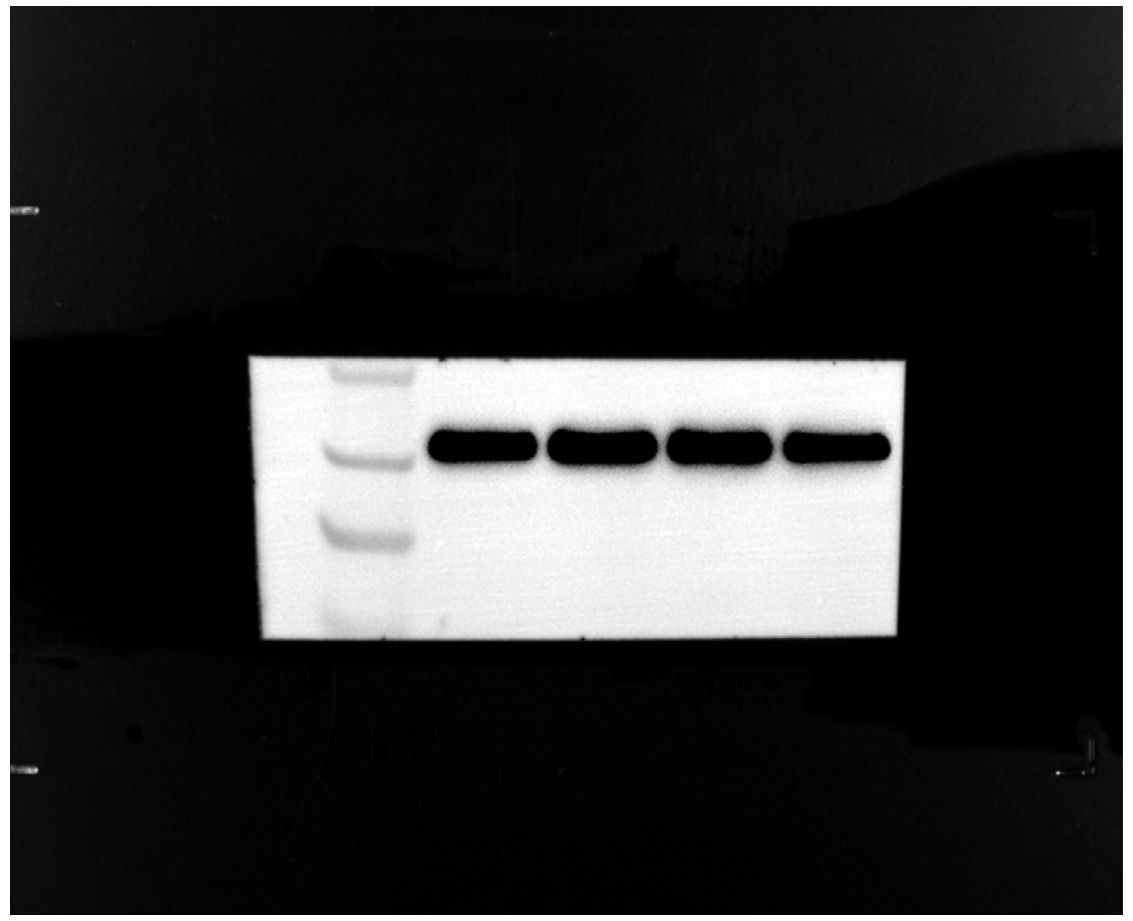

β-actin-1-786-o

Supplementary Figure S6. Full uncropped Western blot images for EMT- and invasion-related proteins in Caki-2 cells.

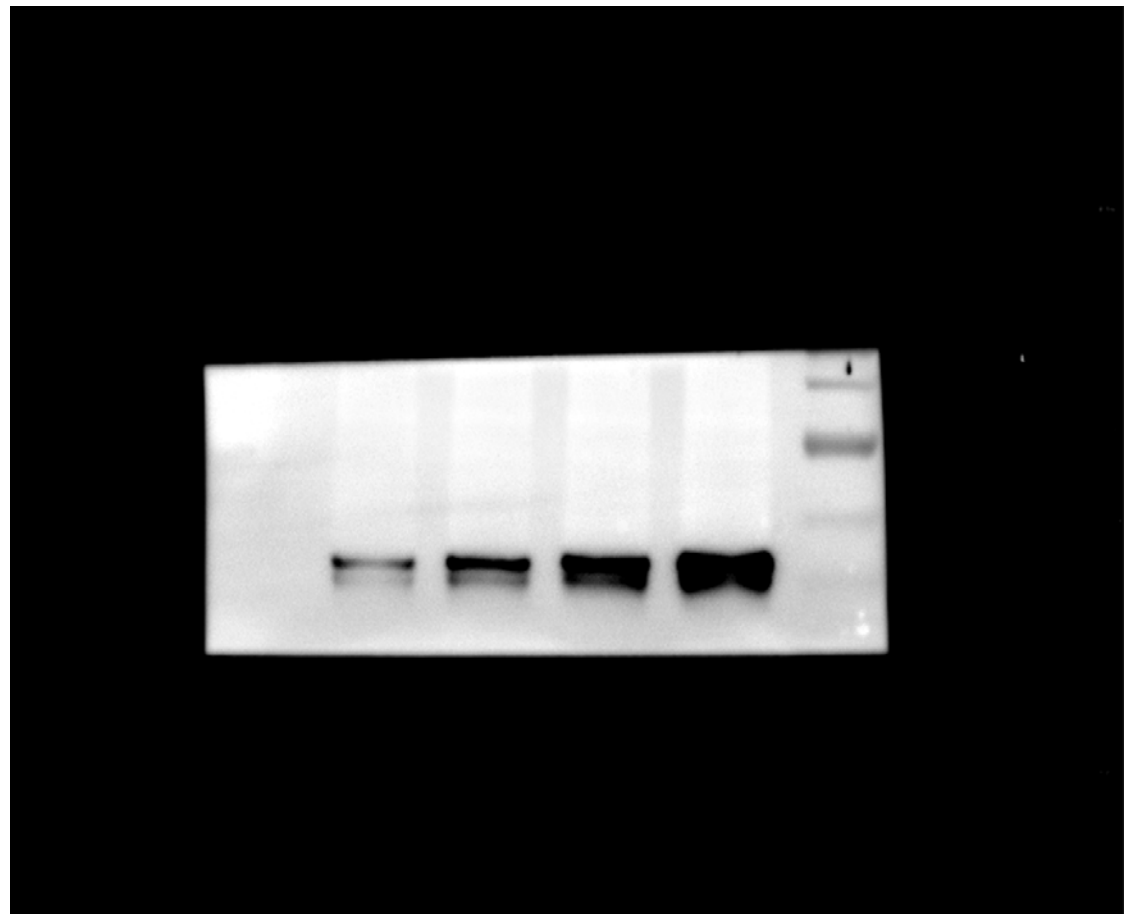

E-cadherin-1-caki2

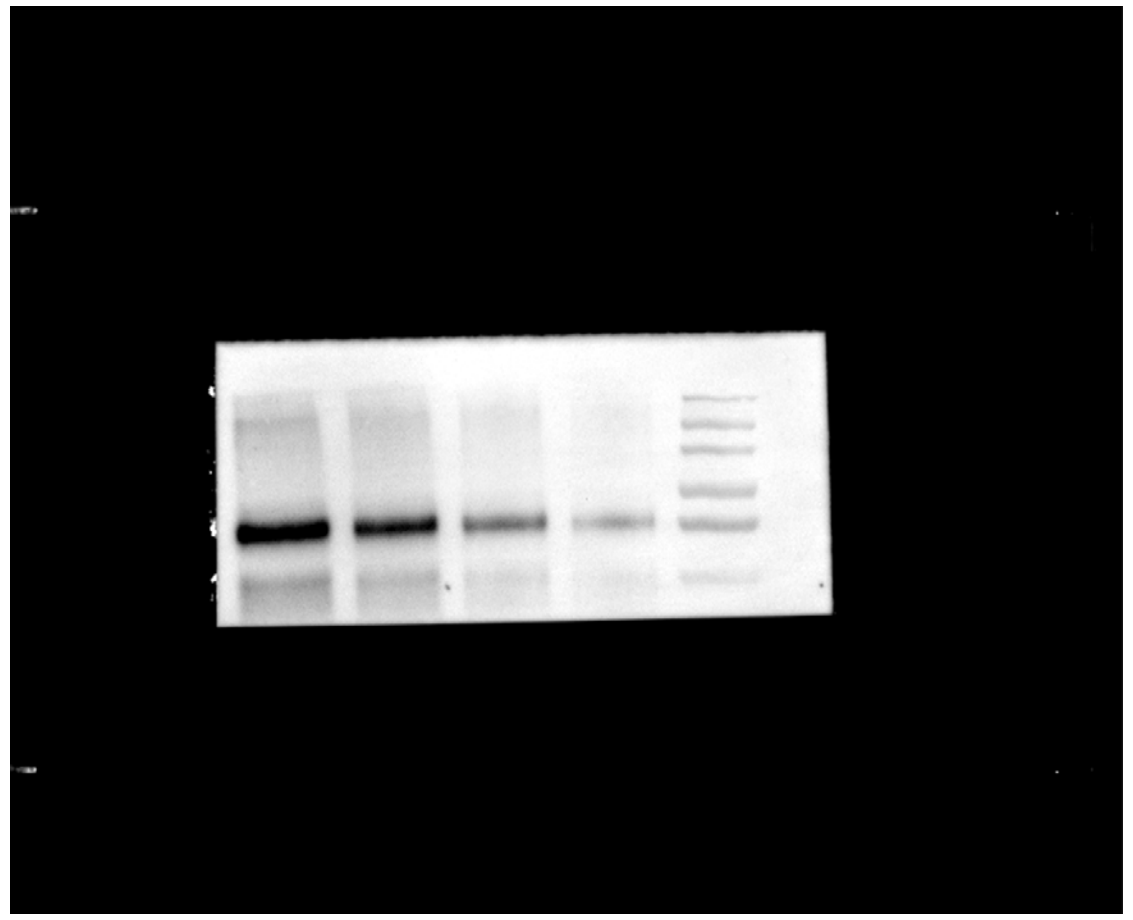

MMP9-1-caki2

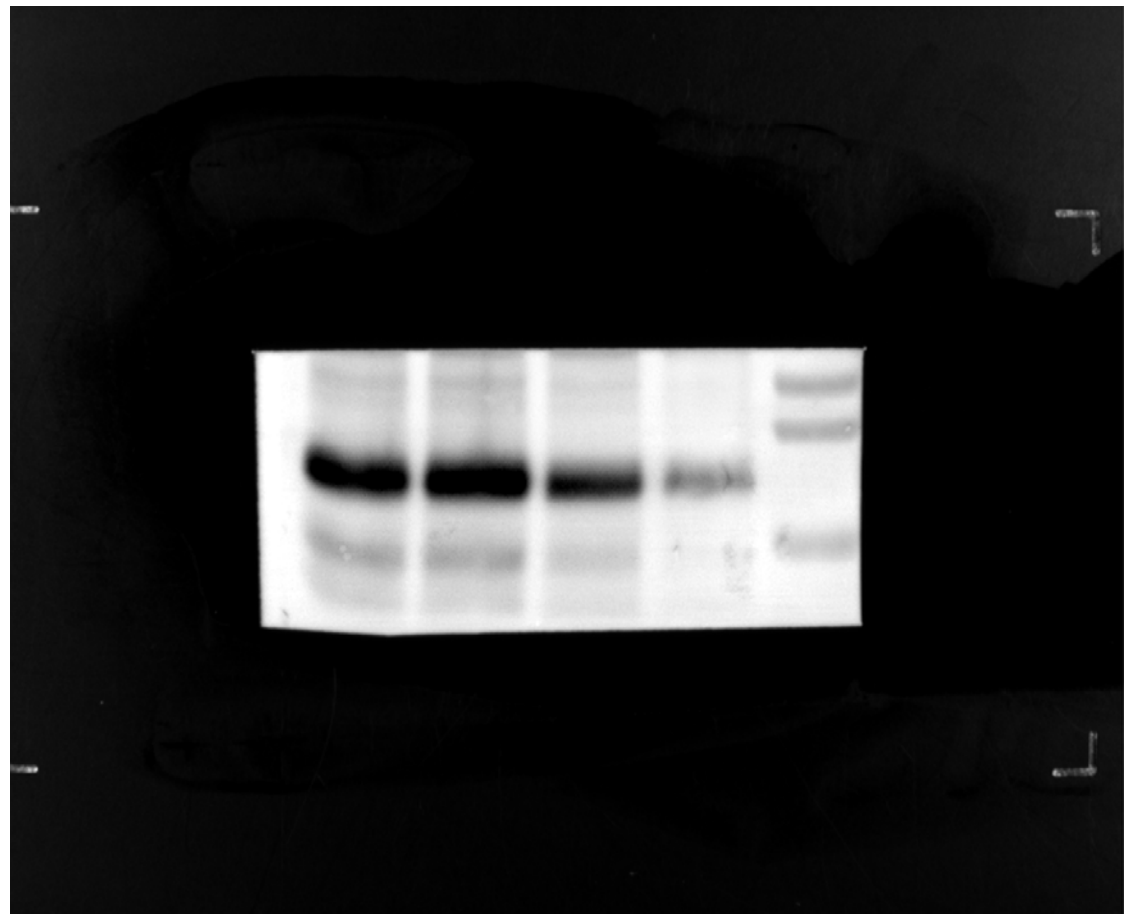

N-cadherin-1-caki2

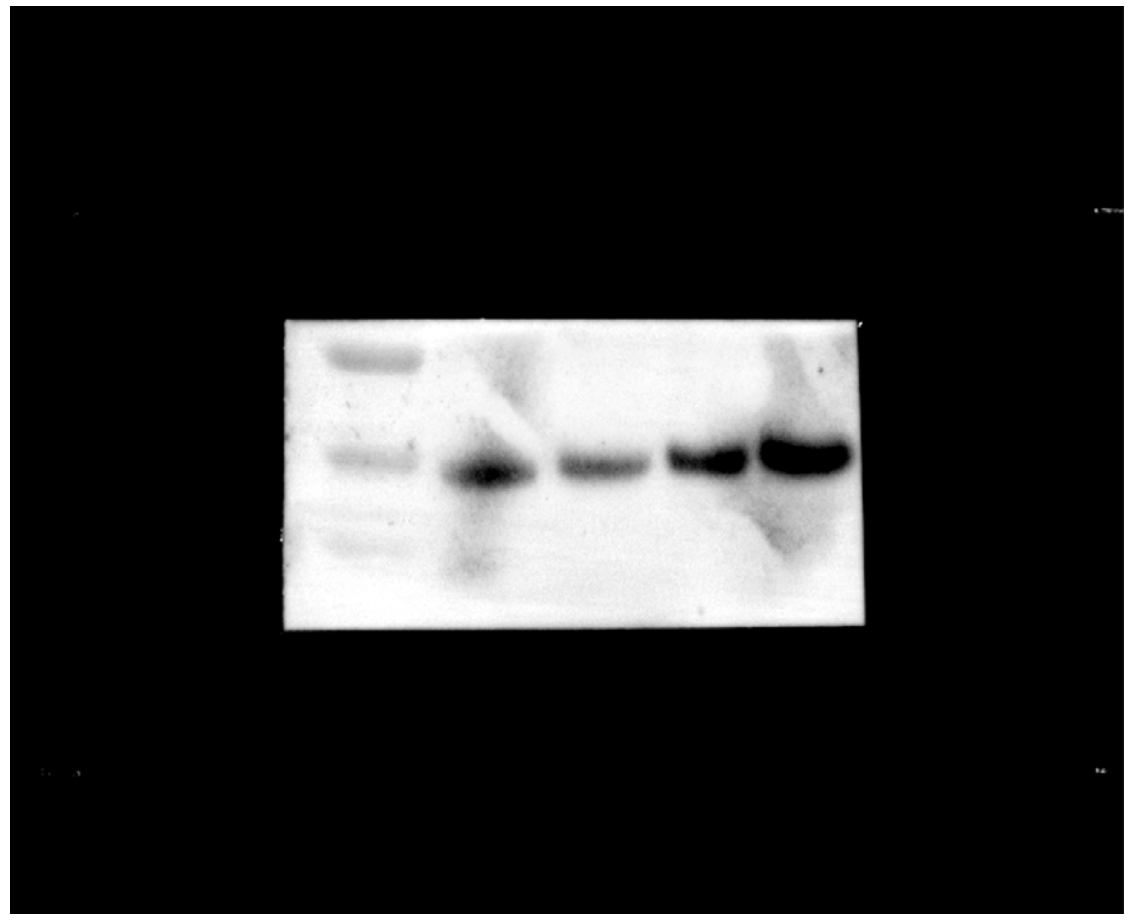

TIMP2-1-caki2

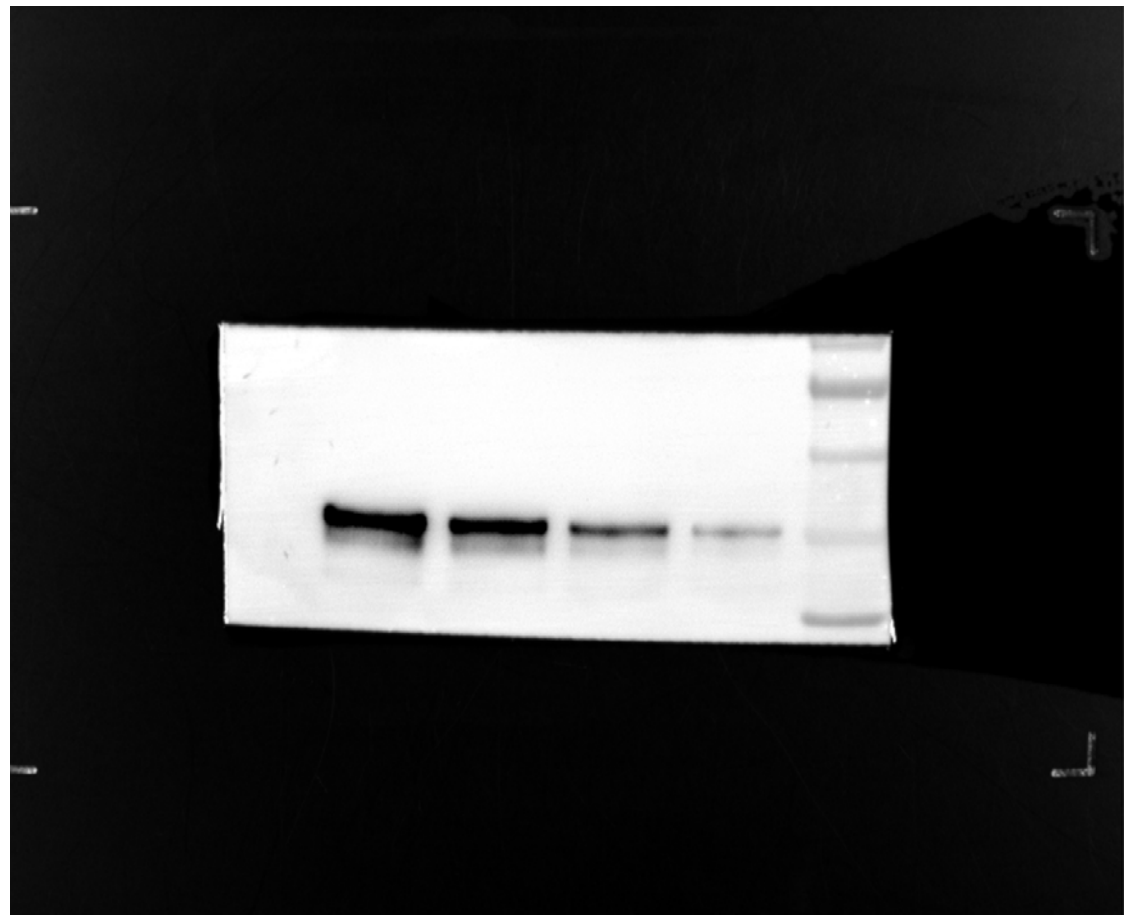

Vimentin-1-caki2

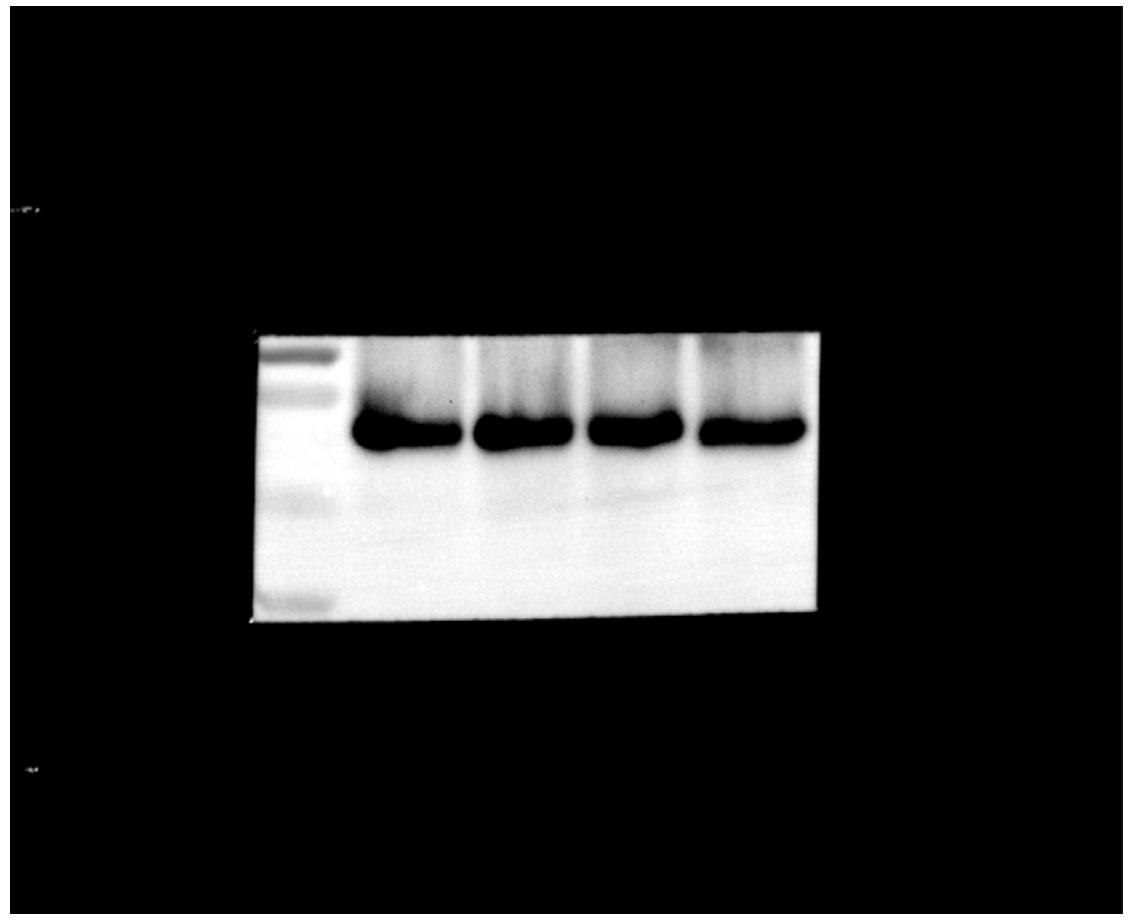

β-actin-1-caki2

Lane 1: 0 μM propranolol; Lane 2: 10 μM propranolol; Lane 3: 25 μM propranolol; Lane 4: 50 μM propranolol. M: protein molecular weight marker.
